# Diversity of hydrolases from hydrothermal vent sediments of the Levante Bay, Vulcano Island (Aeolian archipelago) identified by activity-based metagenomics and biochemical characterization of new esterases and an arabinopyranosidase

**DOI:** 10.1007/s00253-015-6873-x

**Published:** 2015-08-13

**Authors:** Antonio Placido, Tran Hai, Manuel Ferrer, Tatyana N. Chernikova, Marco Distaso, Dale Armstrong, Alexander F. Yakunin, Stepan V. Toshchakov, Michail M. Yakimov, Ilya V. Kublanov, Olga V. Golyshina, Graziano Pesole, Luigi R. Ceci, Peter N. Golyshin

**Affiliations:** Institute of Biomembranes and Bioenergetics (CNR), Via Amendola 165/A, 70126 Bari, Italy; School of Biological Sciences, Bangor University, Bangor, Gwynedd LL57 2UW UK; Institute of Catalysis, Consejo Superior de Investigaciones Científicas (CSIC), 28049 Madrid, Spain; Department of Chemical Engineering and Applied Chemistry, University of Toronto, Toronto, Ontario M5S 3E5 Canada; Immanuel Kant Baltic Federal University, 236040 Kaliningrad, Russia; Institute for Coastal Marine Environment CNR, 98122 Messina, Italy; S.N. Winogradsky Institute of Microbiology, Russian Academy of Sciences, 117312 Moscow, Russia

**Keywords:** Vulcano Island, Fosmids, Metagenomic library, Screening, Hydrolase, Lipase, Esterase, Arabinopyranosidase

## Abstract

A metagenomic fosmid expression library established from environmental DNA (eDNA) from the shallow hot vent sediment sample collected from the Levante Bay, Vulcano Island (Aeolian archipelago) was established in *Escherichia coli*. Using activity-based screening assays, we have assessed 9600 fosmid clones corresponding to approximately 350 Mbp of the cloned eDNA, for the lipases/esterases/lactamases, haloalkane and haloacid dehalogenases, and glycoside hydrolases. Thirty-four positive fosmid clones were selected from the total of 120 positive hits and sequenced to yield ca. 1360 kbp of high-quality assemblies. Fosmid inserts were attributed to the members of ten bacterial phyla, including *Proteobacteria*, *Bacteroidetes*, *Acidobateria*, *Firmicutes*, *Verrucomicrobia*, *Chloroflexi*, *Spirochaetes*, *Thermotogae*, *Armatimonadetes*, and *Planctomycetes*. Of ca. 200 proteins with high biotechnological potential identified therein, we have characterized in detail three distinct α/β-hydrolases (LIPESV12_9, LIPESV12_24, LIPESV12_26) and one new α-arabinopyranosidase (GLV12_5). All LIPESV12 enzymes revealed distinct substrate specificities tested against 43 structurally diverse esters and 4 *p*-nitrophenol carboxyl esters. Of 16 different glycosides tested, the GLV12_5 hydrolysed only *p*-nitrophenol-α-(l)-arabinopyranose with a high specific activity of about 2.7 kU/mg protein. Most of the α/β-hydrolases were thermophilic and revealed a high tolerance to, and high activities in the presence of, numerous heavy metal ions. Among them, the LIPESV12_24 was the best temperature-adapted, retaining its activity after 40 min of incubation at 90 °C. Furthermore, enzymes were active in organic solvents (e.g., >30 % methanol). Both LIPESV12_24 and LIPESV12_26 had the GXSXG pentapeptides and the catalytic triads Ser-Asp-His typical to the representatives of carboxylesterases of EC 3.1.1.1.

**Electronic supplementary material:**

The online version of this article (doi:10.1007/s00253-015-6873-x) contains supplementary material, which is available to authorized users.

## Introduction

The genomic and metabolic diversity of prokaryotic domains of life is an extraordinary source for the development of innovative bio-based products of a high application value (Erickson et al. 2012; Buschke et al. 2013; Cragg and Newman 2013; He et al. 2014; Yu 2014). Marine environments contain an exceptional biodiversity generated and supported by a diversified range of special substrates and extreme conditions, such as high and low temperatures, extreme pH values, elevated salinities, pressure, and even irradiation. The microbes capable of growth under such extreme physico-chemical and nutritional conditions, the extremophiles, are of a special interest for biotechnology (Glöckner and Joint 2010; Zhang and Kim 2010; Levin and Sibuet 2012). Among extreme marine habitats, the hydrothermal vents outflowing in volcanic areas are inhabited by thermophilic and hyperthermophilic microorganisms (Zusuki et al. 2004). The latter represent a source of novel thermoresistant enzymes with many outstanding properties (Sellek and Chaudhuri 1999; Bruins et al. 2001; Atomi 2005; Kubalov et al. 2009; Wemheuer et al. 2013). The enzymes from extremophilic microorganisms, the extremozymes, become increasingly attractive for modern biotechnology, especially in bio-conversion of waste and renewable substrates for producing biochemicals, and alternative energy (Nogi and Kato 1999; Ferrer et al. 2005; Poli et al. 2010; Blunt et al. 2014; Elleuche et al. 2014; López-López et al., 2014; Grawe et al. 2015). Since extremozymes also exhibit high resistances to solvents, detergents, and high pressure (Alcaide et al. 2014), they became the catalysts of choice in different industrial bio-processes (Egorova and Antranikian 2005). In particular, the hydrolytic enzymes such as carboxylesterases (EC 3.1.1.1), lipases (EC 3.1.1.3), and cellulases (EC 3.2.1.4) are attractive biocatalysts for a number of commercial applications in food, laundry, pharmaceutical, and other chemical industries (Bornscheuer 2002; Frazzetto 2003, Panda and Gowrishankar 2005; Kennedy et al. 2011).

The Levante Bay is situated on the northeastern side of the Vulcano Island and has an active hot gas vent field at a depth of less than 1 m (Frazzetta et al. 1984). The above sampling site selection therefore fulfils the following important criteria: physical and chemical stability, salinity, elevated temperature, and variety of electron donors and acceptors, determining diverse microbial metabolisms, which makes it a valuable site for metagenomic enzyme study. Moreover, it was shown earlier that some sites of the Vulcano Island hosted dozens of new genera of cultured bacteria and archaea (White et al. 2008). In this work, we report the results of our initial activity-based survey of the metagenomic expression library generated from the environmental DNA (eDNA) extracted from the above site. We characterized three carboxyesterases and one glycosyl hydrolase with respect to their substrate specificities and biochemical properties, and presented the details on the two novel thermostable and solvent-resistant carboxylesterases (E.C. 3.1.1.1) from the family α/β-hydrolase-6, which also exhibited a robust activity in buffers containing polar organic solvents and metal ions.

## Materials and methods

### Site description and sample collection

The sediments (each of approx. 300 g wet weight) were collected at the Levante Bay (Vulcano Island, Aeolian archipelago) (38.4162° N, 14.9603° E) on October 2, 2012, at the depth of water column of about 0.5–2.0 m; the sediment depth was 0–0.3 m.

### Bacterial strains and vectors used in this work

The *Escherichia coli* strains applied in this research for the construction of a metagenomic fosmid library as well as for cloning and protein expression, and the primer pairs for cloning the most significant hydrolases are shown in Table 1. Luria-Bertani (LB) liquid broth and LB agar media were used to grow *E. coli*. Antibiotics applied in media were chloramphenicol (12.5 mg/l) for fosmids and ampicillin (100 mg/l) for pET-46c Ek/LIC expression vectors cloned in *E. coli*.

**Table 1:** Bacterial strains and vectors used for this research

Strain, vector, or primers	Relevant markers and characteristics	Reference
*Escherichia coli* EPI300	The cells contain an inducible mutant *trfA* and *tonA* genotype: *F-mcrA Δ*(*mrr-hsdRMS-mcrBC*) *φ80dlacZΔM15 ΔlacX74 recA1 endA1 araD139 Δ*(*ara*, *leu*)*7697 galU galK Γ- rpsL nupG trfA tonA*	Epicentre, Madison, WI, USA
*E. coli* NovaBlue	endA1 hsdR17 (r_K12_^−^ m_K12_^+^) supE44 thi-1 recA1 gyrA96 relA1 lac F′[proA^+^B^+^ lacI^q^ZΔM15::Tn10] (Tet^R^)	Novagen, Merck, Germany
*E. coli* BL21(DE3)	*fhuA2* [*lon*] *ompT gal* (*λ DE3*) [*dcm*] ∆*hsdS λ DE3* = *λ sBamHIo* ∆*EcoRI-B int*::(*lacI*::*PlacUV5*::*T7 gene1*) *i21* ∆*nin5*	Biolab, CA, USA
pCC2FOS-vector	A copy control fosmid kit (Cat. No. CCFOS059)	Epicentre, USA
pET46c/Lic	Ligase-independent cloning vector kit Ek/LIC cloning kit of Novagen (Cat. No. 71335)	Novagen, Merck, Germany
*E. coli* NovaBlue	Transformant strain harbouring LIPESV12_24	This study
*E. coli* BL21(DE3)	Protein expression strain harbouring LIPESV12_24	This study
E. coli NovaBlue	Transformant strain harbouring LIPESV12_26	This study
*E. coli* BL21(DE3)	Protein expression strain harbouring LIPESV12_26	This study
*E. coli* NovaBlue	Transformant strain harbouring ABO_1197	This study
*E. coli* BL21(DE3)	Protein expression strain harbouring ABO_1197	This study
*E. coli* NovaBlue	Transformant strain harbouring ABO_1251	This study
*E. coli* BL21(DE3)	Protein expression strain harbouring ABO_1251	This study

### Extraction of eDNA from the Vulcano sediment samples and generation of the fosmid library

Extraction of eDNA from 10 g of the sediments was done using the protocol of Meta-G-Nome DNA Isolation Kit (Epicentre Biotechnologies; WI, USA). The quality and sizes of the extracted DNA were evaluated using agarose electrophoresis, and the DNA concentration was estimated with Quant-iT dsDNA Assay Kit (Invitrogen). DNA fragments of 30–40 kb after the end repair were recovered using electrophoresis in a low-melting-point agarose gel, extracted using the Gelase (Epicentre Biotechnologies; WI, USA) and ligated to the linearized fosmid vector pCC2FOS according to the protocol of the manufacturer (CopyControl™ Fosmid Library Production Kit, Epicentre). After the in vitro packaging into the phage lambda (MaxPlax™ Lambda Packaging Extract, Epicentre), the transfected phage T1-resistant EPI300™-T1^R^*E. coli* cells were spread on LB agar medium containing 12.5 μg/ml chloramphenicol and incubated at 37 °C overnight to determine the titre of the phage particles. The resulting library dubbed V12 had a total titre of 40,000 clones. The ten randomly chosen fosmid clones were analysed using *Not*I and/or *Bam*HI endonuclease digestion of purified fosmids to evaluate the size of the cloned eDNA. For longer-term storage, the whole library was plated onto the same solid medium, and after an overnight growth, colonies were collected from the agar surface by using liquid LB medium containing 20 % (*v*/*v*) sterile glycerol and the aliquots were stored at −80 °C.

### Screening the metagenomic library V12

Single fosmid clones obtained by plating the pooled library from the above step on LB agar were arrayed in 384-microtitre plates containing LB medium supplemented with chloramphenicol. The cells were grown at 37 °C overnight and then directly used for screening assays. Replica plates were also produced and stored at −80 °C in the LB broth with chloramphenicol (12.5 μg/ml) and 20 % (*v*/*v*) glycerol for next using.

### Agar-based esterase/lipase activity screening

Every six master 384-well microtitre plates were printed on the surface of a large (22.5 cm × 22.5 cm) square Petri dish containing LB medium supplemented with chloramphenicol (12.5 mg/l), copy control fosmid induction solution (Epicentre Biotechnologies; Madison, USA) (2 ml/l), and 0.5–1.0 % (*v*/*v*) tributyrin emulsified with gum arabic (2:1, *v*/*v*) by sonication. The replicated clones were grown for 18–40 h at 37 °C. The active lipolytic enzymes hydrolysed tributyrin and formed transparent zones around the colonies. The active esterase hits were verified by using Fast Blue RR and α-naphthyl acetate solution, as described previously (Khalameyzer et al. 1999).

### Screening for cellulase activities

The cellulolytic active hits were identified by using low-density carboxymethyl cellulose (CMC) with a final concentration of 0.5 % weight to volume (*w*/*v*). After 48 h of growth at 37 °C, the colonies were thoroughly removed from the agar plates by small volumes of 0.1 % (*w*/*v*) Congo Red (CR) solution in 20 % (*v*/*v*) ethanol. Then, the agar plates were submerged in the new CR solution and stained for 20 min with shaking. The unbound CR was removed from the plates by washing with 1 M NaCl two times, each for 30 min. The cellulose-active clones formed transparent zones around the colonies. The activity confirmation in selected clones was done in *ø* 9-cm Petri dishes.

### Screening for other glycoside hydrolase activities

The glycosidase activity screening was carried out by using chromogenic glycosides, such as 5-bromo-4-chloro-3-indolyl-β-d-galactopyranoside (X-gal) for beta-galactosidases and other substrate-non-specific glycosidases (30 μg/ml), in which the actively expressed enzymes hydrolyse the glycosidic bonds and turned the colourless substrate into insoluble indigo-like blue precipitates. The rescreening was done in mini agar plates (*ø* 6 cm) by using the fluorescent aglicon of 4-methylumbelliferyl beta-(d)-glycoside.

### Screening for haloalkane dehalogenases and haloacid dehalogenases

Clones were replicated into 96-well microtitre plates containing per well 200 μl of LB broth with chloramphenicol and the induction solution (as above), and grown overnight (18 h) at 37 °C with shaking at 220 rpm. Then, 20 μl of reaction cocktail of haloacids and haloalkanoic acids (chloroacetic acid, bromoacetic acid, 1,3-dichloropropanol, 1,2-dibromopropanol, 3-bromo-2-methylpropionic acid) at a concentration of 0.1 mM each in 10 mM *N*-(2-hydroxyethyl)piperazine-*N*′-(3-propanesulphonic acid) (EPPS) buffer pH 8.0, containing 20 % (*v*/*v*) of dimethylsulphoxide, was added. Sealed plates were incubated at 37 °C with shaking at 180 rpm for further 30 min. Then, 5 μl of 0.5 mM phenol red in the EPPS buffer was added to the vials and positive hits were detected by the production of a yellow colour due to the liberation of inorganic haloacids into the medium. The activity was re-confirmed on agar plates. Overnight-grown colonies were covered with a soft (0.6 %) agar in the EPPS buffer containing the substrate cocktail (4 % *w*/*v*). After a short incubation (30 min) at 37 °C, the hits were verified by adding the pH indicators.

### Extraction of fosmid DNA for sequencing

The fosmid DNA extraction was done by using the Large-Construct Kit (QIAGEN, Hilden). The host chromosomal DNA contamination in the samples was reduced by using ATP-dependent exonuclease of Epicentre (Cambio Ltd., Cambridge, UK). The purity of the fosmid DNA and the assessment of the approximate size of the cloned fragment were done by *Not*I and/or *Bam*HI endonuclease digestion and fragments’ visualization after agarose gel electrophoresis.

### DNA sequencing, insert assembly, and annotation

The Sanger reads for terminal nucleotide stretches of each purified fosmid were done by Macrogen Ltd. (Amsterdam, The Netherlands). Then, the pools of the individual fosmids were sequenced using the Illumina MiSeq platform. For the preparation of libraries for next-generation sequencing, we pooled five fosmids in a minicentrifuge tube in equimolar ratios. DNA fragmentation was conducted with Bioruptor UCD-200 sonicator (Diagenode, Belgium) using parameters adjusted to obtain 800–1000-bp fragments. The fragment libraries have been prepared by NebNext Ultra DNA Library preparation kit (New England Biolabs, USA) according to the manufacturer’s instructions. Then, the multiplexed libraries were sequenced on MiSeq Sequencing System (Illumina, San Diego, USA) with a 2 × 150-bp sequencing kit.

Obtained paired end reads were subjected to stringent quality filtering and trimming by CLC Genomics Workbench 6.5 (CLCbio, Denmark), and removal of reads associated to *E. coli* K12 genomic DNA and pCC2FOS vector DNA was performed by the Deconseq software package (Schmieder and Edwards 2011). Reads remaining after the filtering step were used for de novo assembly with a CLC assembler and further scaffolding by SSPACE software (Boetzer et al. 2011) and in silico filling of gaps using GapFiller (Boetzer and Pirovano, 2012). Assembled contigs were checked for quality using mapping of all the reads back to contigs and analysis of uniformity of coverage and distribution of broken read pairs along the contig. Completeness of insert sequences of a contig was confirmed by occurrence of pCC2FOS polylinker sequences. To identify fosmids in the pool, we initially performed Sanger sequencing of the termini of each fosmid using standard pCC2FOS sequencing primers (Epicentre, UK) and aligned resulting reads with all contigs obtained for a pool using a local BLAST algorithm.

Gene prediction and primary functional annotation were done using the RAST annotation pipeline (Aziz et al. 2008; Overbeek et al. 2014) and the MetaGeneMark annotation software (http://opal.biology.gatech.edu). The taxonomy of the hosts was analysed also by using BLAST2GO software (Conesa et al. 2005). Amino acid alignment was done by using ClustalW2 as well as HMMER tools (http://hmmer.janelia.org, Finn et al. 2011), and the phyla and orders were predicted using an E value < e^−20^ as a cut-off. The multiple protein alignments were conducted also by using the MUSCLE application (Edgar 2004) and ClustalW in BioEdit software (Hall 1999) with default settings. The neighbour-joining and maximum likelihood trees were constructed in MEGA v.6.06 (Tamura et al. 2013) using the settings for the Poisson model and homogenous patterning between lineages. The bootstrapping was performed with 1000 replicates, if not indicated otherwise. The 3D structure prediction for LIPESV12_24 and LIPESV12_26 was analysed using Phyr_2 (http://www.sbg.bio.ic.ac.uk/phyre2) and Pymol (www.pymol.org).

### Gene cloning and protein purification

For characterization of enzymes predicted in the positive fosmid clones, we have chosen three carboxylesterases and one glycosyl hydrolase. Their genes were amplified by PCR using MyTaq™ Red DNA polymerase (Bioline, London, UK) and the custom oligonucleotide primer pairs: LIPESV12-9-FP and RP; LIPESV12_24-FP and RP; LIPESV12_26-FP and RP; ABO_1197-FP and RP; ABO_1251-FP and RP; and GLV12_5-FP and RP. The oligonucleotide sequences with pET-46c Ek/LIC adaptors are given in Table 2. The corresponding positive fosmid was used as a template to amplify the target genes.

**Table 2:** Primers were designed and used in this study

Primers^a^	Oligosequences of direction 5′ to 3′
LIPESV12-9-FP	GACGACGACAAGATGCGGTACCTGAATGAAGTG
LIPESV12-9-RP	GAGGAGAAGCCCGGTTATTTAAAAAAAGACTTC
LIPESV12_24-FP	GACGACGACAAGATGACCATCACCACCAGCGAAAG
LIPESV12_24-RP	GAGGAGAAGCCCGGTTAACTTGAGGCGGGCGGGG
LIPESV12_26-FP	GACGACGACAAGATGCCGCACCCCACCATCCAGAC
LIPESV12_26-RP	GAGGAGAAGCCCGGTTACGATTTGCTGGAAGAGAC
ABO_1197-FP	GACGACGACAAGATGCAACTGAAACACCTTTTTC
ABO_1197-RP	GAGGAGAAGCCCGGTTAGGGGCGAACTTCGCGCCAGC
ABO_1251-FP	GACGACGACAAGATGATGACAGCAATAATTCGTC
ABO_1251-RP	GAGGAGAAGCCCGGTTAAACCACCGGGATGATGTC
GLV12_5-FP	GACGACGACAAGATGCCTGTGAAGAACGTCCTTC
GLV12_5-RP	GAGGAGAAGCCCGGTTACCGGAAATCCAGTTCGTAC

^a^The oligomers were designed with adapter after Ek/LIC Cloning Kit instruction of Novagen (Merck, Germany) and were purchased from Eurofins (Eurofins Genomics, Ebersberg, Germany)

### Cloning and expression of selected genes in *E. coli*

The PCR primer pairs (Table 2) with the nucleotide adapters used in this research were designed following a ligation-independent cloning protocol of Novagen (Darmstadt, Germany). The reactions were done in a Techne TC-5000 cycler (CA, USA) using the following programme: 1 cycle of 95 °C/3 min following 35 cycles of 95 °C for 30s, 55 °C for 30s, and 72 °C for 1 min per 1000 nucleotides, followed by one extension at 72 °C for 5 min. The purified PCR products were then purified, treated with an endonuclease, annealed to *His-tag* harbouring the pET-46c Ek/LIC vector, and transformed into *E. coli* NovaBlue according to the protocol of the manufacturer (Novagen, Darmstadt, Germany). The DNA inserts in the resulting plasmids were verified by sequencing services of Macrogen Ltd. (Amsterdam, The Netherlands).

The expression of hydrolases in *E. coli* BL-21 (DE3) was carried out in two steps: at first, the inocula were grown in LB medium supplemented with 100 μg/l ampicillin in an incubator at 37 °C, with shaking at 220 rpm to the OD600 of 0.8–0.9. The cultures were then transferred to an incubator at 18 °C and induced by adding isopropyl-β-d-galactopyranoside (IPTG) at a final concentration of 0.5 mM. The cells were grown overnight under the above conditions and then harvested by centrifugation (5000*g* for 30 min at 4 °C). The recombinant proteins were purified by affinity chromatography on Ni-NTA His-Bind columns (Novagen) and gel filtration using 10-kDa cut-off centrifugal filter units (Merck KGaA) according to the protocol of the manufacturers. The size and purities of the his-tagged protein preparations have been analysed by sodium dodecyl sulphate polyacrylamide gel electrophoresis (SDS-PAGE) using 10 % *v*/*v* precast gels (Expedeon).

### Hydrolytic activity assay

The general assay methods were reported previously (Gupta et al. 2002). In our study, the hydrolytic activities of the LIPESV12 α/β-hydrolases were (if not mentioned otherwise) estimated colorimetrically in 500 μl of 50 mM potassium phosphate (K,P) buffer (pH 7.0), containing 0.1 % Triton X-100, substrate, and enzyme as described recently (Pérez et al. 2012; Tchigvintsev et al. 2014). Briefly, the substrates *para*-nitrophenyl (*p-*NP)-carboxylic acid esters were added from 10 mM stock solution in a buffer containing acetonitrile:dimethyl sulphoxide (1:1 of *v*/*v*) to get a final concentration of 1 mM. The enzyme (1.8 μg) for reaction (native) or for reference (overheated) was added from stock solution after 1 min of preincubation of the reaction vials at an appropriate temperature as requested. The reactions (if not mentioned otherwise) were incubated in an Eppendorf thermomixer comfort with a mixing frequency of 500 rpm. After 10 min of incubation, if not indicated otherwise, the reactions were stopped by adding 500 μl of cold K,P-buffer (50 mM, pH 8.0) containing 10 mM EDTA (pH 8.0). The stopping solution did change the pH in the reaction vial, at the values tested, to slightly alkaline (pH 7.2–8.0) for maintaining the released *p*-NP in the phenolate form. The absorption was measured on a spectrophotometer of model JENWAY 6300 (Progen, UK), if not described otherwise, at 410 nm in the temperature range from 10 to 50 °C or at 380 nm (55–80 °C) with respect to hypsochromic shift and blunting peak formation of the overheated *p*-NP. The blank samples with all reaction components and with inactivated enzymes were run in parallel. Before reading the absorbance of each enzyme reaction, the absorbance of the blank was set to 0, in order to omit the background rates caused by random hydrolysis of the *p*-NP-ester substrates during incubation. Then, the concentration of enzyme products was calculated using simple linear regression equation (Microsoft Excel) given on each individual standard curve of *p*-NP (Sigma-Aldrich, UK, PA grade) for each test series. The substrate profiling assays for other hydrolytic activities against esters other than *p*-NP esters were tested in 96-well plates using 43 structurally diverse esters (read at 540 nm) as described recently (Alcaide et al. 2015). Briefly, assay reactions (in triplicate) were conducted at 30 °C measuring the absorbance during a total time of 30 min each 1 min in a Synergy HT Multi-Mode Microplate Reader (BioTek, Bad Friedrichshall, Germany). Reaction mixture started by adding 1.3 μg protein stock solution to an assay mixture containing 4 μl of ester stock solution (200 mg/ml in acetonitrile), to a final concentration of 4 mg/ml, in 196 μl of 5 mM EPPS buffer, pH 8.0, containing 0.45 mM phenol red (*ε* at 540 nm = 8500 M^−1^ cm^−1^).

Glycosidase activity for GLV12_5 was assayed using 15 different *p*-NP sugar derivatives that included α/β-glucose, α/β-galactose, α-maltose, β-cellobiose, α/β-arabinopyranose, α/β-arabinofuranose, α/β-xylose, β-mannose, α-rhamnose, and α-fucose in 96-well plates as previously described (Alcaide et al. 2015). Briefly, assay reactions were conducted at 30 °C by adding 1 μg enzyme and 10 μl of substrate from a stock solution (10 mg/ml freshly prepared in 45 mM 4-(2-hydroxyethyl)-1-piperazineethanesulphonic acid (HEPES) buffer, pH 7.0) in 190 μl of 45 mM HEPES buffer, pH 7.0, to get a final volume of each vial of 200 μl, and the final substrate concentration was 500 μg/ml. Absorbance was read in triplicate assays at 405 nm in a Synergy HT Multi-Mode Microplate Reader (BioTek, Bad Friedrichshall, Germany) to follow the extent of the hydrolysis (*ε*_*p*-NP_ at 405 nm = 16,325 M^−1^ cm^−1^) during 30 min. All values were corrected with non-enzymatic transformation as blank.

The *p*-NP-butyrate (C4) is visually more stable relating to diverse temperatures and pH values of buffers as well as in the presence of metal ions or organic solvents in comparison to *p*-NP-acetate (C2). Therefore, we used *p*-NP-C4 in this study as a universal substrate for biochemical enzyme characterization. Thermal stability was determined after incubation of the enzyme for 5, 20, 30, and 40 min at temperatures 55, 60, 70, 80, and 90 °C with shaking at 500 rpm. The enzyme solutions were cooled down on ice, and the activity was estimated following the standard protocol as described in the “Materials and methods”. The half-life for LIPESV12_24 was measured by incubating the enzyme at 55 °C, and samples were taken after 0.5, 1, 2, 3, 4, and 24 h. Then, the retaining activities were assayed using 1 mM *p*-NP-C4 as substrate.

The optimal pH for the enzyme activity was determined in the range of pH 5.5–9.0 at 30 °C in 20 mM of different buffer systems including sodium citrate (pH 5.5), potassium phosphate (pH 7.0), HEPES (pH 7.5), Tris-HCl (pH 8.0), and Tris-HCl (pH 9.0), with incubation time of 10 min. The substrate for the assays was 1 mM *p*-NP-C4. The reference variant with inactivated enzymes was applied and used also as blank for absorbance reading. The enzyme reactions were stopped by adding the buffer with higher pH values for maintaining *p*-NP released in a phenolate form as described above. The concentration of released *p*-NP was determined using an individual standard curve for each experiment as described above.

The effects of different bivalent cations (Cu^2+^, Mn^2+^, Mg^2+^, Zn^2+^, Co^2+^, and Ca^2+^) each at 1 mM, and organic solvents (acetonitrile, methanol, ethanol, isopropanol, 1,2-propanediol, and dibutyl phosphate) in a range of concentrations from 5 to 80 % (*v*/*v*) on the activity of the LIPESV12 proteins were also evaluated. All the enzyme tests were performed in triplicate at 30 °C/10 min using 1 mM *p*-NP-C4 as substrate as mentioned above. The average values with standard deviations were applied.

In all cases, the specific activity of enzymes were given in units per milligram of protein, where one unit (U) of activity was defined as the amount of enzyme required to transform 1 μmol of substrate in 1 min under the assay conditions.

### Chemicals

All chemicals used for enzymatic tests were of PA grade, which have been purchased from Sigma-Aldrich Chemical Co. (St. Louis, MO, USA). The kits applied in this study with individual manufacturers have been noticed in the text.

### Accession numbers

The genes cloned in this study for LIPESV12_9, LIPESV12_24, LIPESTV12_26, and GLV12_5 are deposited in the EMBL/GenBank/DDBJ databases under the accession numbers KR919661, KP861227, KP861228, and KR919662, respectively.

## Results

### V12 fosmid library generation and screening the industrial relevant enzymes

As estimated by the library titration and insert restriction analysis of a set of randomly chosen fosmids, the V12 library contained approx. 40,000 clones with cloned DNA fragments between 30 and 40 kbp in size, which implies the V12 library size corresponds to approximately 1.4 Gbp of the cloned eDNA.

Using agar plates containing one of the following substrates, tributyrin, carboxylmethylcellulose, mixture of haloacids and haloalkanoic acids, or X-gal, we screened 9600 clones and identified 120 positive hits. Among them, 50 hits were positive for the hydrolysis of tributyrin showing carboxylesterase/lipase activities, 36 hits for the hydrolysis of X-gal showing β-glycosyl hydrolase (galactosidase) activities, 10 hits for the hydrolysis of carboxylmethylcellulose (CMC) showing cellulase activity, and 24 hits revealed dehalogenase activities.

**Fig. 1:**
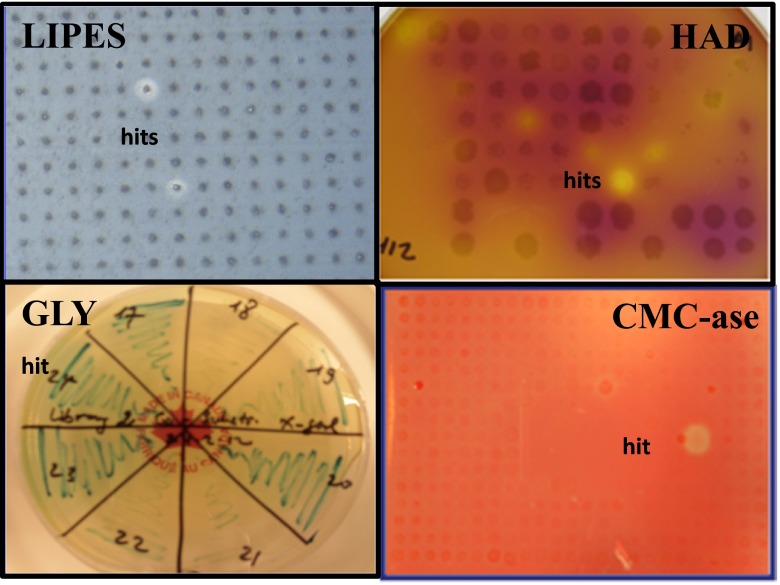
Agar-based activity screens of lipolytic enzymes (*LIPES*), haloalkane dehalogenases (*HAD*), glycosidases (*GLY*), and cellulases (*CMC-ase*) from the Vulcano fosmid library. The halo zones produced by hits are indicated. Among them, the HAD hits, as mentioned in the “Materials and methods”, have been rescreened from microtitre plate-based activity screening

Among the 120 hits, a set of 40 most active clones, which revealed larger halo zones on agar plates containing tributyrin (lipases/esterases (LIPES) relating hits) and cellulases (CMCases) or strong chromogenic signals of glycosidase (GLY) and dehalogenases (HAD), were chosen for further analysis (some representative hits are presented on Fig. 1). The termini of purified fosmids were Sanger-sequenced by using standard pCC2-FOS forward and reverse primers. The Sanger read analysis allowed us to filter redundant hits or those with high similarity with already-characterized proteins or with absolute nucleotide identities with known genomes. In that manner, six hits including four HADs, one LIPES, and one GH were discarded from further analysis.

### DNA sequence analysis of hits

The total size of the eDNA corresponding to the selected 34 hits was about 1360 kbp. By the annotation of the sequences, we have identified about 200 open reading frames (ORFs) encoding relevant hydrolytic enzymes of interest. In fact, most of them showed only partial domain similarities to hydrolases from non-redundant databases. We have chosen 60 ORFs encoding esterases/lipases (26 hits) (EC 3.1.1.-) and β-lactamases (9 hits) (EC 3.5.2.-), and 5 ORFs encoding haloalkane and haloacid dehalogenases (HAD) (EC 3.8.1.-), C-N hydrolases (5 hits) (EC 3.5.-.), and glycosyl hydrolases (EC 3.2.1.-) (15 hits) (Supplementary Table S1). Among the annotated ORFs, the β-lactamases, as well as some thioesterases and the C-N hydrolase genes, were deduced from the nucleotide sequences of the hits exhibiting tributyrin hydrolytic activities as described in the “Materials and methods” section.

### Phylogenetic analysis of hydrolytic enzymes

**Fig. 2:**
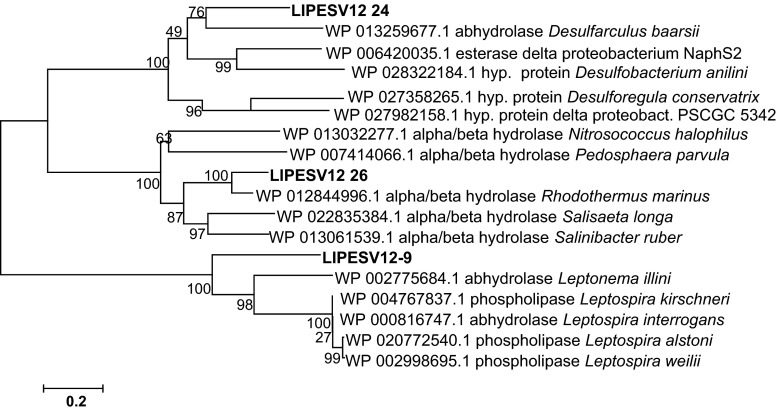
Neighbour-joining phylogenetic tree of the carboxylesterases LIPESV12-9, LIPESV12_24, and LIPESV12_26 and their closely clustered enzymes. The multiple protein alignment was conducted using the MUSCLE application (Edgar, 2004) and BioEdit software (Hall, 1999) with default settings. The phylogenetic neighbour-joining trees were constructed using MEGA v.6.06 (Tamura et al., 2013) as described in the “Materials and methods” with 100 bootstrap replicates. The *scale bar* reflects the number of substitutions per position

From BLAST analysis carried out using the selected 60 ORFs as query sequences, we have identified the proteins with the highest identity and putative producing organisms. The 60 hydrolases were affiliated within a broad spectrum of bacterial phyla to include *Proteobacteria* (E value ranging from 1.00e^−35^ to 0), *Bacteroidetes* (5.00e^−48^ to 0), *Acidobacteria* (1.00e^−128^–0), *Firmicutes* (2.00e^−22^–5.00e^−127^), *Verrucomicrobia* (5.00e^−22^–9.00e^−114^), *Chloroflexi* (0), *Spirochaetes* (1.00e^−99^), *Thermotogae* (1.00e^−164^), *Armatimonadetes* (1.00e^−50^), and *Planctomycetes* (1.00e^−156^). Among only the lipolytic and dehalogenase hits, 18 associated to known and three to unknown bacterial orders. For more details about the nearest microbial taxa from which cloned DNA fragments were derived, see Tables S1 and S2 in the Electronic supplementary material (ESM). The protein families within each hydrolase group, namely, the LIPESV12_series for lipases/esterases (EC 3.1.1.-), the BLAV12_series for β-lactamases (EC 3.5.2.-), the C-NV12_series for nitrilases and other C-N hydrolases (EC 3.5.-.-), the HADV12_proteins of haloalkane and haloacid dehalogenases (EC 3.8.1.-), and the GLV12_series for glycosyl hydrolases (EC 3.2.1.-), have been established using interactive sequence similarity searching and HMMER software and is presented in Supplementary Table 2. The clan-specific phylogenetic positions of the lipases/esterases (LIPESV12_), 9 beta-lactamases (BLAV12_), and 5 C-N hydrolases (C-NV12_), in lipolytic positive hits have been also highlighted in Figs. S1, S2, and S3 of the ESM, respectively. The relationship between five putative HADV12_proteins and their nearest counterparts is presented in Fig. S4. As the HADV12_3 and HADV12_4 were related to α/β-hydrolase superfamily including trehalose phosphatases, the other HADV12_proteins clustered nearly to their counterparts from *Desulfarculus baarsii* and *Microbulbifer agariliticus* (Fig. S4). The phylogenetic analysis for 15 GLV12_proteins is also illustrated in Supplementary Fig. S5. The phylogenetic position of GLV12_5 enzyme, one of the glycosyl hydrolases identified with the largest halo zone on CMC (Fig. 1) with 37 other closely clustered proteins. As a result of the phylogenetic analysis, we have chosen a set of significant new hydrolases, particularly three LIPESV12 (Fig. 2) and one GLV12 enzymes, for further biochemical characterization.

### Biochemical characterization of His-tagged proteins

#### Substrate specificity of LIPESV12_enzymes

After verifying the nucleotide sequences by the Sanger readers, we purified three 6His-tagged LIPESV12 proteins (LIPESV12-9, LIPESV12_24, LIPESV12_26) and one glycosyl hydrolase (GLV12_5) to homogeneity using Ni-NTA affinity chromatography and ultrafiltration with 10 kDa cut-off. The relative molecular masses of the purified protein preparations were confirmed by SDS-PAGE and are illustrated in Fig. S6. For the biochemical characterization, we applied different *p-*NP esters such as *p-*NP-C2, *p-*NP-C4, *p-*NP-laurate (C12), and *p-*NP-palmitate (C16). The standard enzyme tests have been done as described in the “Materials and methods”. As references, we have chosen two carboxyesterases, ABO_1177 and ABO_1521, which were characterized recently (Tchviginsev et al. 2014). All of the LIPESV12 hydrolases under standard assay conditions showed higher activities against the *p*-NP-acetate in comparison to the referent ABO enzymes, which preferred the *p*-NP-C4 as optimal substrate (Table 3). The hydrolytic activities of LIPESV12 enzymes against long-chain fatty acid *p*-NP-esters were significantly lower, even not detectable for LIPESV12-9 and ABO_1251 even after 50-min incubations. After 50 min of reaction under the conditions described in the “Materials and methods”, the other three, LIPESV12_24, LIPESV12_26, and ABO_1197, were significantly activated also in the hydrolysis of the long-chain ester *p*-NP-C16 and released approx. 128.0, 554.0, and 12.0 μmol *p*-NP per mg protein, respectively. Further, to study the substrate specificities of the LIPESV12 enzymes with respect to other esters with diverse chain lengths and stereo-configurations in more detail, we performed the substrate fingerprinting, with the results presented below.

**Table 3:** Substrate specificities, as maximum activities, of the LIPESV12 enzymes in comparison to ABO esterases as references

Enzymes/substrate^a^	*p*-NP-C2	*p*-NP-C4	*p*-NP-C12^b^	*p*-NP-C16^b^
LIPESV12_9	1145.5 ± 5.3	334.4 ± 2.3	0	0
LIPESV12_24	960.0 ± 0.5	392.4 ± 0.6	42.1 ± 1.2	127.6 ± 0.6
LIPESV12_26	1053.4 ± 1.7	806.0 ± 0.5	314.4 ± 2.7	553.5 ± 1.7
ABO_1197	61.3 ± 6.6	254.7 ± 1.8	110.4 ± 7.3	10.2 ± 12.6
ABO_1251	650.1 ± 4.5	2100.0 ± 0.3	57.4 ± 5.8	0

^a^Enzyme reactions were carried out as described in the “Materials and methods”. The *p*-NP-esters were added from 10 mM stock solution of each to get an end concentration of 1 mM. The enzyme assays were set up in triplicate

^b^The incubation time prolonged to 50 min. The specific activities corresponding to units per milligram of protein with standard deviation (±SD) rounded to decimal were given. Protein concentration here and hereafter was estimated by using the standard Bradford reagent kit B-6916 from Sigma

#### Substrate fingerprinting for the representative LIPESV12 enzymes

**Fig. 3:**
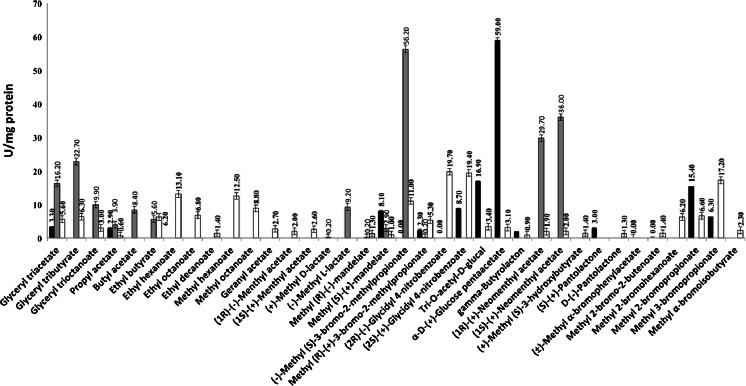
Substrate profiles of the enzymes with a set of 43 structurally diverse compounds. The specific activities were calculated in triplicate, and average values with standard deviation are shown. The specific esterase/lipase activity of each enzyme (in units/mg protein) against a set of structurally diverse esters was measured after incubation at 30 °C each minute within 30 min using 1.3 μg protein at pH 8.0 in 5 mM EPPS buffer and 4 mg/ml esters. Of the total 43 compounds, the LIPESV12-9 (*solid bar*) hydrolysed 17 esters, LIPESV12_24 (*grey bar*) hydrolysed 15 esters, and LIPESV12_26 (*open bar*) hydrolysed 33 esters. Some of the activity values (for the cases of ethyl myristate, ethyl benzoate, methyl decanoate, methyl oleate, (R)-(+)-glycidol, (S)-(-)-glycidol, and gama-valerolactone) were negligible and we rounded to 0

A total of 43 ester-like chemicals, other than *p*-NP esters, were used to evaluate the substrate ranges and specific activities (U/mg) of the three LIPESV12 enzymes at pH 8.0 and at 30 °C (Fig. 3). Under our assay conditions, the three ester hydrolases hydrolysed short-to-medium-chain-length tri-acyl-glycerides and alkyl esters, but with different ranges of reactivities and orders of preference. Thus, substrate fingerprints revealed that V12_26 (33 positive substrates) exhibited the widest substrate range. Among the three enzymes, V12_26 did show more lipase character, as compared to V12_24 and V12-9; this was demonstrated by its higher capacity to hydrolyse methyl and ethyl hexanoate and octanoate and ethyl decanoate. Halogenated esters were also hydrolysed by V12-9 and V12_26, with the latter being the only one acting against halogenated esters with double bonds (i.e. the alkenyl halogenated ester methyl 2-bromo-2-butenoate). Under our assay conditions, all three ester hydrolases were also found to be enantio-selective to different degrees and preferences for at least eight chiral esters, including methyl-(±)-mandelate, methyl-(±)-lactate, (±)-menthylacetate, (±)-neomenthyl acetate, (±)-glycidyl 4-nitrobenzoate, (±)-pantolactone, and (±)-methyl (*S*)-3-hydroxybutyrate. Three hydrolases further utilized tri-*O*-acetyl-glucan and two the carbohydrate ester α-d-glucose pentaacetate (Fig. 3).

#### Influence of temperature on stability and activity of LIPESV12_24

Since the V12 library was generated from a hydrothermal site, we expected LIPESV12 enzymes to exhibit some habitat-specific features. Indeed, not all of the purified enzymes exhibited their thermophilic nature. The maximal activity for activity of LIPESV12_24 was at about 70–80 °C (∼2800 and ∼3980 U/mg), while LIPESV12_26 reached its maximum activity at 50 °C (∼1312 U/mg). A summary of the enzyme activity vs temperature profile is shown in Table 4.

**Table 4:** Temperature profiles of the V12 α/β-hydrolases

	Activity (%)^b^	
*T* (°C)^a^	LIPESV12_24	LIPESV12_26
10	62.0 ± 0.5	20.0 ± 0.5
20	57.0 ± 0.5	72.0 ± 0.5
30	100^c^	100^e^
37	406.0 ± 0.5	116.0 ± 1.0
45	451 ± 0.5	200.0 ± 1.0
50	517 ± 0.5	258.0 ± 0.1
60	100^d^	87.0 ± 0.5^e^
70	127.0 ± 0.5	n.d
80	173.0 ± 0.5	n.d

^a^Enzyme reactions were carried out in triplicate as described in the “Materials and methods” by using *p*-NP-butyrate (1 mM) as substrate

^b^The measurements of absorbance in the range 10–50 °C were conducted at the wavelength 410 nm and at 60–80 °C—at 380 nm, since the heated *p*-NP revealed a hypsochromic shift as mentioned in the “Materials and methods”, and the results with ±SD are given, where 100 % activity of ^c^359.0 ± 0.5, ^d^2219.0 ± 0.5, and ^e^524 ± 1.0 U/mg of proteins

**Fig. 4:**
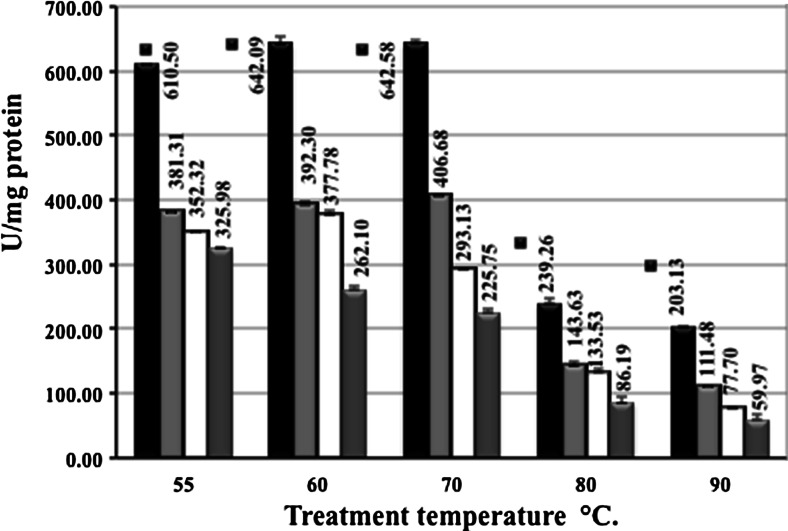
The thermostability of LIPESV12_24 as a function of incubation time. Since the LIPESV12_24 showed a broad temperature profile and actively hydrolysed the substrates at high temperatures (see Table 3), we checked its thermostability at different incubation times (5, 10, 30, and 40 min) and temperatures of 55, 60, 70, 80, and 90 °C. Residual activity was detected using standard test at 30 °C as described in the “Materials and methods” section. The *activity values given on the top of each column* were corresponded also to incubation time: after 5 (*solid bar*), 20 (*light-grey*), 30 (*dashed grey*), and 40 (*open bar*) min using 1 mM *p-*NP-butyrate

The thermostability profiles of LIPESV12_24 are shown in Fig. 4. Noteworthy, the protein manifested a robust residual activity at temperatures ranging from 55 to 90 °C. After 5 and 10 min of incubation at 60–70 °C, the activity was highest and decreased after 40 min of incubation. After a short incubation at 90 °C (for 5 min), the enzyme revealed a lower yet significant activity (∼ 203 U/mg) pointing at its thermostability. The enzyme became inactive only after a longer incubation time (40 min, 90 °C). The half-life of LIPESV12_24 at 55 °C was also estimated by incubating the enzyme at the temperature and withdrawing aliquots for measurement after 0.5, 1, 2, 3, 4, and 24 h by the manner described in the “Materials and methods”. The LIPESV12_24 exhibited about 60 and 40 % its maximum activity (100 % activity corresponded to 610 ± 0.5 U/mg) after 3 and 4 h of incubation. Therefore, its half-life was estimated as being approx. 3–3.5 h.

### Effects of pH and bivalent cations on the activity of enzymes

**Fig. 5:**
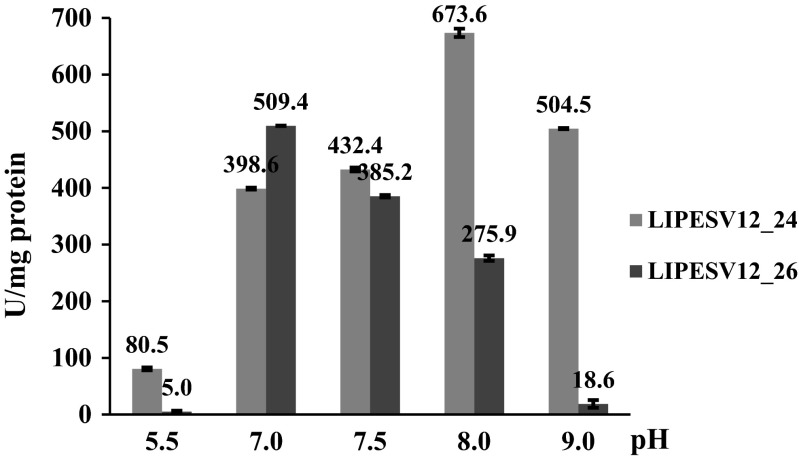
Hydrolytic activities of LIPESV12_24 and LIPESV12_26. LIPESV12_24 against *p-*NP-butyrate at different pH values. A wide pH spectrum of the enzyme activities was demonstrated. The data indicates that the enzymes were neutrophilic or slightly alkaline

We have stopped the enzyme reaction by adding cold alkaline buffer containing EDTA (pH 8.0) in order to quench the released *p*-NP into *para*-nitrophenylate form allowing the colorimetric measurement of the latter at a standard wavelength of 410 nm. These measurements were usefully applied to the pH activity optimum determination. The pH range was established at 5.5, 7.0, 7.5, 8.0, and 9.0 by applying citrate, phosphate, HEPES, and Tris-HCl buffers as described in the “Materials and methods”. Both the LIPESV12_24 and LIPESV12_26 enzymes altered significantly the activities at acidic pH values: under slightly acidic conditions (pH 5.5), the activity of LIPESV12_24 decreased for about 25 % while the LIPESV12_26 was fully inactivated (remained <1 %) in comparison to their activities at pH 7.0. Both enzymes were particularly active at slightly alkaline (pH 8.0) and neutral (7.0) pH values (Fig. 5). The inhibitory effect was observed also for the LIPESV12_26, even at pH 8.0. On the contrary, the LIPESV12_24 retained about 75 % of its activity at pH 9.0 in comparison to its highest values (Fig. 5).

**Fig. 6:**
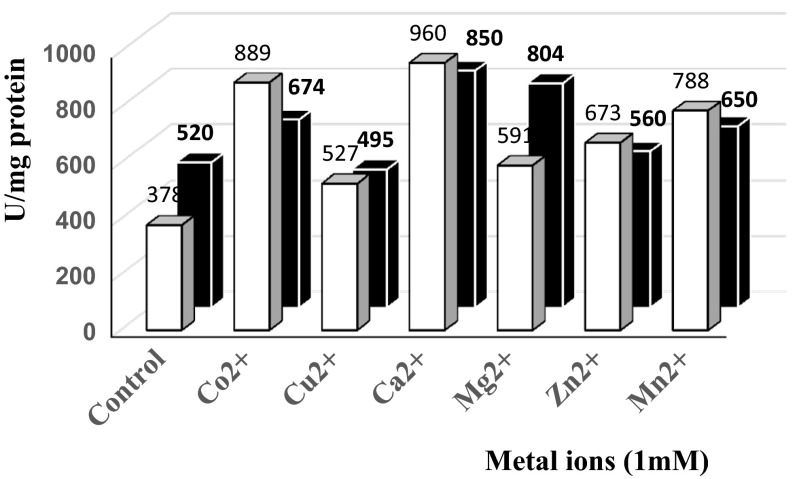
Influences of bivalent metal ions: Cu^+2^, Co^+2^, Zn^+2^, Mn^+2^, Mg^+2^, and Ca^+2^ on the activity of LIPESV12_24 (*solid bar*) and LIPESV12_26 (*grey bar*). The reaction mixture in each vial was preincubated at 30 °C in the presence of 1 mM bivalent cation for 5 min. Then, the substrate (1 mM of *p*-NP-butyrate) was added and activity was measured under standard conditions as described in the “Materials and methods”. Both enzymes were activated by the addition of cations: Co^2+^, Ca^2+^, and Mn^2+^ stimulated activities twofold to threefold towards the substrate in comparison to the control variants. After a longer incubation period (50 min), activities decreased, and in the case of Cu^2+^ and Mn^+2^, the activity of LIPESV12_24 dropped from ∼527 and ∼788 U/mg (5 min of incubation) to ∼21 and ∼73 U/mg (50 min of incubation), respectively

The enzyme kinetics may also be influenced by cofactors such as metal ions. Volcanic environments are characterized by the presence of metal ions at elevated concentrations. We measured the influence of bivalent cations such as Cu^2+^, Co^2+^, Zn^2+^, Mn^2+^, Mg^2+^, and Ca^2+^ on the activity of purified LIPESV12_24 during short-term (5 min) and long-term (50 min) incubations. After 5 min of the exposure, the enzyme activity was increased in comparison to the control. Reaction mixtures containing calcium, cobalt, and magnesium salts, each of 1 mM, showed almost a twofold increase in the activities as compared to the control (Fig. 6). Long incubation of the enzyme in metal ions in most cases seemed to be not result the increase of enzyme activities. In opposite, some inhibitory effects of copper, zinc, and manganese were observed after a long-term incubation (data not shown). The common effects of the divalent metal ions on the activities of the LIPESV12_24 and LIPESV12_26 enzymes are clearly consistent with the metal-rich conditions in the environment of origin of the source microorganisms.

### Effect of organic solvents

We analysed the activity of both enzymes using *p-*NP-butyrate in the buffer supplemented at different concentrations with 1,2-propanediol, isopropanol, ethanol, methanol, acetonitrile, and dibutyl phosphate, the main component of degradation products of tributyl phosphate (TBP), the extractant used in the nuclear fuel reprocessing known as the PUREX process (Table 5). By adding to 5 % (*v*/*v*) dibutyl phosphate in the potassium phosphate buffer (pH 7.0), the specific activities of both, LIPESV12_24 and LIPESV12_26, enzymes were strongly stimulated (∼2600 and ∼955 U/mg, respectively). Interestingly, both LIPESV12_enzymes were activated by acetonitrile with a final concentration of 30 % (*v*/*v*) (∼1746 and 954.0 U/mg, respectively). Although to a lesser extent than in dibutyl phosphate and acetonitrile, LIPESV12_ proteins revealed a high specific activity in different alcohols, such as 30 % of 1,2-propanediol (∼1039 U/mg for LIPESV12_24 and ∼592 U/mg for LIPESV12_26) and 10 % ethanol (∼500 and ∼542 U/mg, respectively) (Table 5). We have tested also ethanol of concentration up to 30 % (*v*/*v*), but no activation effects have been observed (data not shown). The LIPESV12_24 enzyme displayed the following activities in methanol: ∼685, ∼945, and ∼769 U/mg in buffer supplemented with 10, 20, and 30 % (vol/vol), respectively. At higher concentrations of methanol, the LIPESV12_24 still revealed a significant activity: 126 U/mg at 40 % and 127 U/mg at 50 % (*v*/*v*). The similar profile showed also the LIPESV12_26, which in the presence of 30 % (*v*/*v*) methanol had activity of ∼914 U/mg (Table 5). Both LIPESV12 enzymes were also active in 20 % (*v*/*v*) isopropanol and developed significant catalytic activities of ∼732 and ∼341 U/mg, respectively. The obtained data indicated the metagenome-derived new α/β-hydrolases were capable of retaining their activities in different polar protic and aprotic solvents.

**Table 5:** Hydrolytic activity of LIPESV12_24 and LIPESV12_26 in solvents

KP buffer (50 mM) supplemented with	Solvent added (%, *v*/*v*)	Spec. act. in U/mg (protein)^a^	
		LIPESV12_24	LIPESV12_26
KP buffer	None	360.0 ± 0.1	524.0 ± 0.5
Dibutyl phosphate	5	2600.0 ± 0.5	955.0 ± 0.5
Acetonitrile	30	1746.0 ± 0.5	954.0 ± 0.1
1,2-Propanediol	30	1039.0 ± 0.5	592.0 ± 1.0
Methanol	30	769.0 ± 0.3	914.0 ± 1.0
Isopropanol	20	732.0 ± 0.1	341.0 ± 1.0
Ethanol	10	500.0 ± 0.5	542.0 ± 0.1

^a^Enzyme reactions were carried out as described in the notes of Tables 3 and 4. The enzyme assay was set up in triplicate, and the results ± SD are given in the table

Moreover, we have determined also some kinetic parameters such as Michaelis constant (Km) for the LIPESV12 α/β-hydrolases. The concentrations of substrates, either *p-*NP-C3 or *p-*NP-C4, varied between 1 and 40 mM. Under the standard assay conditions, the Km for *p-*NP-C4 was 100 μM for LIPESV12_26 and 10 μM for LIPESV12_24. For the ABO_1197 and ABO_1251, the activity varied as per substrate between 780 μM (*p*-NP-C3) and 5 μM (*p*-NP-C4), respectively, as reported recently (Tchigvintsev et al. 2014).

### Structure prediction analysis for two new hydrolases: LIPESV12_24 and LIPESV12_26

Both HMMER (Supplementary Table S2) and BLAST indicated that the LIPESV12_26 has a high identity (70–81 %) with the proteins WP_012844996.1 and WP_014067971.1 of *Rhodothermus marinus*, while LIPESV12_24 exhibited a significantly lower identity (50 %) with α/β-hydrolases of *D. baarsii*, delta-proteobacterium NaphS2 (42 %), *Desulfatiglans aniline* (39 %), and *Desulforegula conservatrix* (38 %). Meanwhile, the maximum identities to the LIPESV12-9 were found to predict α/β-hydrolase homologues from *Leptonema illini* (WP_002775684.1) of 47 % and from *Leptospira alstoni* (WP_020772540.1) of 42 %. A more detailed phylogenetic analysis for the above findings is presented in Fig. 2.

The putative catalytic triads and the reactive centres of the LIPESV12_24 and LIPESV12_26 with 272 and 275 amino acids, respectively, were analysed by using the Phyre2 and PyMol software available in the abovementioned tools.

The α/β fold of different lipases and esterases contains the typical GXSXG. This motif together with a conserved histidine and aspartate (or glutamate) generates the catalytic triad of α/β-hydrolases (Ollis et al. 1992, Jaeger et al. 1994), which is also present in both V12 hydrolases (see Figs. S7A and B). The possible catalytic triads for LIPESV12_24 and LIPESV12_26 were identified with amino acids S107, D217, and H245, and S94, D218, and H245, respectively. The distances between H245/D217 and H245/D218 in the amino acid sequences of LIPESV12_24 and LIPESV12_26 were 28 and 27 amino acid residues, respectively, similar to the H and D/E distance of about 29 residues reported previously for carboxylic ester hydrolases (Ollis et al. 1992, Jaeger et al. 1994). Based on the three-dimensional model, a shorter distance (3.1 Å) between His245 and Asp217 in the V12_24 in comparison to the distance between His245 and Asp218 in its counterpart reached 5.9 Å (Fig. S7A and B). The polypeptides contain eight α-helix turns and six typical β-sheets, which are also predicted for enzymes of the α/β-hydrolase_6 superfamily.

### Characterization of GLV12_5

**Fig. 7:**
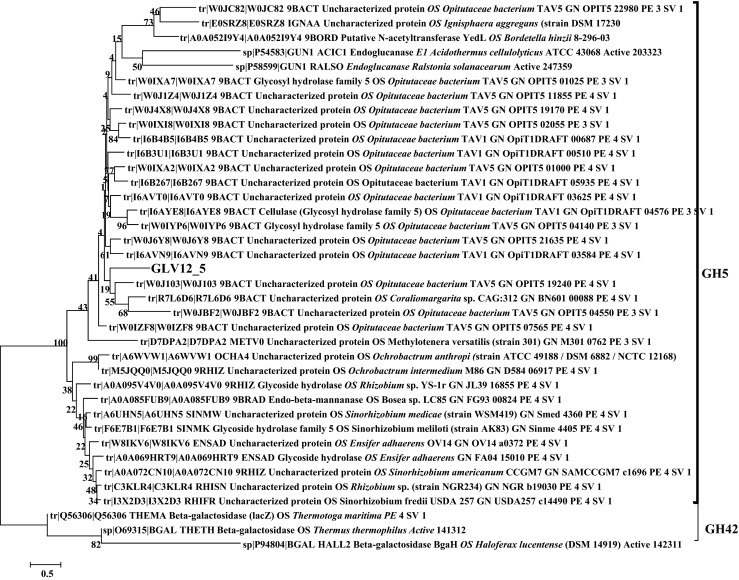
Phylogenetic position of GLV12_5. The conserved functional domains of each protein were determined using Pfam (http://pfam.xfam.org/search) (Finn et al. 2011). The obtained BLAST data sets of the lowest E values were aligned in MEGA6 (Tamura et al. 2013) using MUSCLE (Edgar 2004). The tree was constructed by using the neighbour-joining method (Saitou & Nei 1987) as described in the “Materials and methods”

The glycosyl hydrolase selected for this study, GLV12_5, was derived from a fosmid exhibiting the strongest CMC hydrolysis halo (seen as the largest clearance zone in Fig. 1). The homology as well as the phylogenetic relationship of the GLV12_5 was analysed and presented also in the HMMER electronic supplementary table (Table S2) and in Fig. 7. The new glucosyl hydrolase was clustered with some uncharacterized proteins and also some known cellulases and glycosyl hydrolases from *Opitutaceae* bacterium species (Fig. 7). The PCR product of the gene was cloned using the primer pair GLV12_5-FP and RP with pET46 Ek/LIC adapters, as described in the “Materials and methods” (Table 2). Substrate specificity of the GH enzyme revealed activity towards just one of 16 sugar esters tested, namely, *p*-NP-α-arabinopyranose (see Supplementary Table S3). Under the standard assay conditions, the maximal activity was at 35 °C and pH 5.5 (∼2683 ± 26 U/mg). Therefore, the enzyme should be considered as α-arabinopyranosidase with a quite restricted substrate profile.

## Discussion

The volcanic and geothermal sites are popular subjects for the enzyme biodiversity prospecting, as they harbour microbial communities often composed of extremophiles (Handelsman et al. 1998; Ferrer et al. 2007; Simon and Daniel 2011; Wemheuer et al. 2013; Alcaide et al. 2014). In the present study, we provided a first report on the metagenomic prospecting of hydrolytic enzymes from the shallow hydrothermal vents of the Vulcano Island, Italy, a renowned hot spot of biodiversity of extremophilic microorganisms.

Our results demonstrated that using enzyme activity screening methods, we have successfully obtained numerous hydrolytic enzymes, including lipases and esterases, lactamases/nitrilases, dehalogenases, and glycosyl hydrolases. From DNA sequence analysis of positive fosmid hits, we have predicted and annotated about 200 genes with either full length or with domain homologies to the non-redundant protein databases. From the annotated ORFs, 60 complete proteins deduced have been analysed in more detail. Within this small set of peptide sequences, by performing homology analysis using HMMER and BLAST tools with a low E value cut-off filter, ten known bacterial phyla were predicted as potential hosts of the cloned DNA. The analysis indicated a high taxonomic complexity in the microbial community of the Levante Bay in the Vulcano Island, the enzymatic diversity of which is far from being fully explored. As expected, all affiliated orders were related to marine bacteria, mostly thermophiles from the phyla *Proteobacteria*, *Bacteroidetes*, and *Acidobacteria* (see also Table S1). The organisms donating the eDNA fragments in positive clones were restricted to the organisms typically present in thermophilic, as well as in metal ion- and sulphur-rich milieus. The enzymes studied here in detail, LIPESV12_24, LIPESV12_26, and also LIPESV12-9 and GLV12_5, have been derived from the yet uncultured organisms of the orders *Desulfarculales* and *Incertae sedis* from order II of *Bacteroidetes*, and from the uncharacterized protein of some *Opitutaceae* species (Fig. 2), respectively. The phylogenetic position and homology of both LIPESV12 enzymes with their counterparts from non-redundant protein databases suggested they belong to the α/β-hydrolase_6 (ABHD6) superfamily. The highest similarity, as well as the closest clustering of LIPESV12_24 to the homologous proteins from marine bacteria of the order *Desulfarculales*, and of LIPESV12_26 to the *R. marinus*, and, at the same time, their distant phylogenetic placement one from another (see Fig. 2, Fig. S1, and Table S2), suggests their distinct origin despite a somewhat similar function. Both the LIPESV12 proteins possessed the typical positions of catalytic centres for the carboxylesterases with the conserved triads Ser-His-Asp. The identified numbers of helix turns and beta-sheets indicated the similarity of both enzymes to the carboxylic lipase/esterase group, consistent with the data obtained in the primary polypeptide sequence analysis. But the predicted 3D structures of the LIPESV12 proteins were clearly different from the canonical fold of α/β-hydrolases from other extremozymes such as carboxylic ester hydrolases from hyperthermophiles (Levisson et al. 2009) and the referent proteins in this research: ABO_1197 and ABO_1251 from *Alcanivorax bokumensis* (see also Tchigvinsev et al. 2014). The nearest homologues of LIPESV12_24 are the α/β-hydrolase YP_003808833.1 from *D. baarsii* DSM 2075 (50 % sequence identity), which has been annotated as a lipase (cd00741) with a preference to long-chain esters, and several α/β-hydrolases from the family 6 (ABHD6), which are active in hydrolysis of long-acyl-chain glycerol esters such as arachidonic glycerol ester. In the structural models of LIPESV12_24 and LIPESV12_26, the active sites are located in a deep hydrophobic groove and include the putative catalytic triad residues Ser107/94, His245/245, and Asp217/218, respectively. In both active sites, the catalytic serine residues (Ser107 and Ser94) are located on a sharp turn of the protein backbone structure (the nucleophilic elbow), whereas the interacting residues of both catalytic triads (except for Asp218 in LIPESV12_26) are positioned within hydrogen bonding. This suggests that both proteins use the classical Ser-His-Asp catalytic triad mechanism with serine acting as the nucleophile, histidine as the general acid-base, and aspartate helping to neutralize the charge forming on histidine during the catalysis. These active sites appear to be capable of binding acyl esters with short acyl chain lengths. The structural models also suggest that LIPESV12_26 might have a more open active site that is in line with a broader substrate range of this enzyme compared to LIPESV12_24. Indeed, in our assays, LIPESV12_26 revealed a wider substrate specificity (hydrolysed 33 from 43 esters tested) compared to LIPESV12-9 (7 from 43) and LIPESV12_24 (15 from 43) (Fig. S7A and Fig. S7B). This was confirmed in experiments using *p*-NP-C12 and *p*-NP-C18 as substrates showing the capacity to hydrolyse longer alkyl esters. In contrast to the ABO proteins and LIPESV12-9, after a long incubation, LIPESV12_24 and LIPESV12_26 were able to hydrolyse significant amounts of long-chain fatty acids, including *p-*NP-C12 and *p-*NP-C16.

To this end, the LIPESV12_enzymes are therefore qualified as carboxylesterase EC 3.1.1.1 with wider substrate specificities. The protein engineering and evolution research for these target proteins basing either on naïve error-prone or on direct mutagenesis with more specific databases and tools, such as 3DM (Kourist et al. 2010), have also been planned for our future research. Furthermore, from a biochemical point of view, the enzymes, especially the LIPESV12_24, pointed at a thermophilic nature of their hosts, retaining the activity even after a short incubation at temperatures near the water boiling point.

The enzymes were tolerant to, and active with, different divalent cations such as calcium, cobalt and magnesium, copper, zinc, and manganese. The three latter metals exhibited inhibition on LIPESV12_24 only after a long incubation. The activity of several metal ions is positively influenced at high temperatures possibly due to ionic bond formation, stabilizing the protein molecules.

The pH values have a considerable influence on enzyme kinetics due to the influence on the formation or breaking of electrostatic, hydrogen, and other bonds (Li et al. 2005). The pH profile analysis for the enzymes showed their higher activities in the neutral and slightly alkaline ranges. While the LIPESV12_24 was still active at pH 5.5, the LIPESV12_24 at that pH had almost no activity. This may be explained either by a high buffering capacity of the seawater and thus the selection for neutrophils or by a possible intracellular localization of enzymes.

The use of organic solvents in the industrial biocatalysis offers advantages as compared to the aqueous media due to a higher solubility of hydrophobic substrates, reduced water activity altering the hydrolytic equilibrium, decreased risk of microbial contamination, and others (Sellek and Chaudhuri 1999). Both hydrolases, LIPESV12_24 and LIPESV12_26, could serve as potential candidates for industrial applications, since they kept their stability and activity in organic solvents, even at high concentrations of isopropanol and methanol.

The phylogenetic analysis showed that the representative of GLV12 proteins identified in this study, the GLV12_5, clustered with GH42 and GH5 glycosyl hydrolase groups, which embraced the most cellulose-degrading enzymes. Interestingly, the purified recombinant GLV12_5 protein was highly substrate specific, which revealed activity towards just one of the 16 *p*-NP-glycosides tested, namely the *p*-NP-α-arabinopyranose. This enzyme should be considered as α-arabinopyranosidase (EC 3.2.1_x) with a restricted substrate profile.

The obtained data suggest a high biodiversity of hydrolases in the hydrothermal vents of the Vulcano Island, pointing at a high potential of these environments for mining and exploitation of new industrially relevant enzymes.

## Electronic supplementary material

ESM 1 (PDF 1223 kb)
