# Supplementary material for: Diversity of hydrolases from hydrothermal vent sediments of the Levante Bay, Vulcano Island (Aeolian archipelago) identified by activity-based metagenomics and biochemical characterization of new esterases and an arabinopyranosidase
Source: Appl Microbiol Biotechnol. 2015 Aug 13;99(23):10031–46. doi: 10.1007/s00253-015-6873-x (PMC4643112; doi:10.1007/s00253-015-6873-x)
Supplement: Supplementary file 1 — (PDF 1223 kb) [file 253_2015_6873_MOESM1_ESM.pdf]

Electronic Supplementary Material

Applied Microbiology and Biotechnology

Diversity of hydrolases from hydrothermal vent sediments of the Levante Bay, Vulcano Island (Aeolian archipelago) identified by activity-based metagenomics and biochemical characterisation of new esterases and an arabinopyranosidase

Antonio Placido[1¶], Tran Hai[2¶*], Manuel Ferrer[3], Marco Distaso[2], Tatyana N. Chernikova[2], Dale Armstrong[2], Alexander F. Yakunin[4], Stepan V. Toshchakov[5], Michail M. Yakimov[6], Ilya V. Kublanov[7], Olga V. Golyshina[2], Graziano Pesole[1], Luigi R. Ceci[1] and Peter N. Golyshin[2]

[1]Institute of Biomembranes and Bioenergetics (CNR), Via Amendola 165/A, 70126 Bari, Italy

[2]School of Biological Sciences, Bangor University, LL57 2UW Bangor, Gwynedd, UK

[3]Consejo Superior de Investigaciones Científicas (CSIC), Institute of Catalysis, 28049 Madrid, Spain

[4]Department of Chemical Engineering and Applied Chemistry, University of Toronto, Toronto, Ontario, M5S 3E5, Canada

[5]Immanuel Kant Baltic Federal University, 236040 Kaliningrad, Russia

[6]Institute for Coastal Marine Environment, CNR, 98122 Messina, Italy.

[7]S.N. Winogradsky Institute of Microbiology, Russian Academy of Sciences, 117312 Moscow, Russia.

Corresponding author: Tel.: +44 1248 382566 Fax: +44 1248 382569.

E-mail: t.hai@bangor.ac.uk

Table S1 HMMER alignments for V12- annotated proteins

<sequence id>	<align. start>	<align. end>	<envelop. start>	<envelop. end>	<hmm acc>	<hmm name>	<hmm. start>	<hmm. end>	<hmm. length>	<bit score>	<Indiv. E-value> *)	Cond- E-value>	Signif. database	outcome -peted	<clan>
>BLAV12_1	10	160	7	178	PF00753.22	Lactamase_B	4	180	194	61.66	7.00E-17	1.40E-20	1	0	CL0381
>BLAV12_2	2	162	2	162	PF13483.1	Lactamase_B_3	1	163	163	147.63	2.40E-43	3.20E-47	1	0	CL0381
>BLAV12_3	374	453	362	454	PF00581.15	Rhodanese	12	112	113	68.48	5.70E-19	1.20E-22	1	0	
>BLAV12_4	26	383	16	396	PF00144.19	Beta-lactamase	8	311	330	229.69	4.40E-68	3.00E-72	1	0	CL0013
>BLAV12_5	168	425	150	434	PF00144.19	Beta-lactamase	23	308	330	85.37	3.50E-24	2.40E-28	1	0	CL0013
>BLAV12_6	72	369	60	387	PF00144.19	Beta-lactamase	18	307	330	107.29	7.60E-31	5.10E-35	1	0	CL0013
>BLAV12_7	18	196	17	230	PF00753.22	Lactamase_B	2	159	194	78.97	3.50E-22	9.30E-26	1	0	CL0381
>BLAV12_8	8	200	5	244	PF00753.22	Lactamase_B	4	157	194	65.34	5.20E-18	1.40E-21	1	0	CL0381
>BLAV12_9	38	180	27	181	PF01510.20	Amidase_2	13	131	132	73.5	1.50E-20	1.00E-24	1	0	
>GLV12-1	170	427	166	444	PF04616.9	Glyco_hydro_43	7	257	286	39.43	3.40E-10	9.20E-14	1	0	CL0143
>GLV12-2	330	567	330	568	PF14498.1	Glyco_hyd_65N_2	1	235	236	269.08	3.40E-80	6.90E-84	1	0	CL0103
>GLV12-4	320	601	313	604	PF00128.19	Alpha-amylase	8	312	316	56.34	3.10E-15	6.20E-19	1	0	CL0058
>GLV12-5	418	560	398	584	PF02449.10	Glyco_hydro_42	117	257	374	18.9	0.00064	4.30E-08	0	0	CL0058
>GLV12-6	36	435	20	435	PF01120.12	Alpha_L_fucos	16	347	347	342.88	1.70E-102	2.20E-106	1	0	CL0058
>GLV12-7	28	286	28	287	PF14498.1	Gly-hyd_65N_2	1	235	236	262.21	4.30E-78	2.90E-82	1	0	CL0103
>GLV12-8	220	683	220	683	PF01055.21	Glyco_hydro_31	1	441	441	414.96	3.50E-124	4.70E-128	1	0	CL0058
>GLV12-9	332	758	331	758	PF01055.21	Glyco_hydro_31	2	441	441	417.22	7.20E-125	9.70E-129	1	0	CL0058
>GLV12-10	24	307	23	307	PF04616.9	Glyco_hydro_43	2	286	286	153.33	6.70E-45	1.40E-48	1	0	CL0143
>GLV12-11	117	242	106	244	PF13088.1	BNR_2	134	249	275	17.12	0.0025	6.70E-07	0	0	CL0434
>GLV12-12	72	256	70	257	PF02837.13	Glyco_hydro_2_N	3	166	167	85.2	3.50E-24	7.00E-28	1	0	CL0202
>GLV12-13	52	199	50	208	PF08531.5	Bac_rhamnosid_N	3	161	172	43.25	2.70E-11	3.70E-15	1	0	CL0202
>GLV12-14	88	332	88	387	PF00728.17	Glyco_hydro_20	1	294	351	89.35	2.50E-25	1.70E-29	1	0	CL0058
>GLV12-15	344	633	343	634	PF02836.12	Glyco_hydro_2_C	2	297	298	343.03	1.00E-102	3.40E-106	1	0	CL0058
>GLV12-16	184	441	180	458	PF04616.9	Glyco_hydro_43	7	257	286	39.34	3.70E-10	9.90E-14	1	0	CL0143
>HADV12_1	11	186	10	189	PF13419.1	HAD_2	2	173	176	74.96	8.10E-21	2.70E-24	1	0	CL0137
>HADV12_2	19	194	19	194	PF13419.1	HAD_2	1	176	176	69.62	3.60E-19	9.60E-23	1	0	CL0137
>HADV12_3	36	254	36	257	PF02358.11	Trehalose_PPase	1	232	235	87.81	4.80E-25	6.40E-29	1	0	CL0137
>HADV12_4	5	469	4	470	PF00982.16	Glyco_transf_20	2	473	474	483.7	4.50E-145	6.10E-149	1	0	CL0113
>HADV12_5	14	275	14	275	PF08282.7	Hydrolase_3	1	254	254	150.25	7.10E-44	1.90E-47	1	0	CL0137
>LIPESV12_1	305	352	299	355	PF02272.14	DHHA1	11	65	68	25.84	5.90E-06	4.00E-10	1	0	

Table S1 (continue)

>LIPESV12_2	35	152	34	161	PF00293.23	NUDIX	2	125	135	63.68	1.30E-17	8.60E-22	1	0	CL0261
>LIPESV12_3	171	408	171	409	PF00756.15	Esterase	1	250	251	137.56	4.70E-40	1.90E-43	1	0	CL0028
>LIPESV12_4	30	229	30	230	PF12695.2	Abhydrolase_5	1	144	145	70.25	1.40E-19	1.40E-22	1	0	CL0028
>LIPESV12_5	388	508	387	509	PF01966.17	HD	2	121	122	71.74	4.80E-20	3.60E-23	1	0	CL0237
>LIPESV12-6	4	157	4	160	PF12850.2	Metallophos_2	2	153	156	80.43	1.20E-22	3.10E-26	1	0	CL0163
>LIPESV12_7	7	180	4	182	PF04307.9	DUF457	4	109	157	32.27	5.40E-08	3.60E-12	1	0	CL0368
>LIPESV12_8	27	246	27	247	PF12697.2	Abhydrolase_6	1	227	228	139.91	1.10E-40	8.80E-44	1	0	CL0028
>LIPESV12-9	38	257	38	258	PF12697.2	Abhydrolase_6	1	227	228	139.7	1.30E-40	1.00E-43	1	0	CL0028
>LIPESV12_10	23	256	23	257	PF12697.2	Abhydrolase_6	1	227	228	146.61	9.70E-43	7.20E-46	1	0	CL0028
>LIPESV12_11	66	298	66	299	PF12697.2	Abhydrolase_6	1	227	228	146.27	1.20E-42	1.30E-45	1	0	CL0028
>LIPESV12_12	59	137	59	137	PF03061.17	4HBT	1	79	79	56.76	1.80E-15	2.40E-19	1	0	CL0050
>LIPESV12_13	56	149	42	150	PF00657.17	Lipase_GDSL	142	233	234	35.98	6.20E-09	1.30E-12	1	0	CL0264
>LIPESV12_14	12	144	6	146	PF09500.5	YiiD_Cterm	9	142	144	105.94	1.20E-30	1.60E-34	1	0	CL0050
>LIPESV12-15	18	100	17	102	PF03061.17	4HBT	2	77	79	54.01	1.30E-14	1.70E-18	1	1	CL0050
>LIPESV12_16	75	275	74	276	PF07859.8	Abhydrolase_3	2	210	211	205.74	5.60E-61	2.60E-64	1	0	CL0028
>LIPESV12-17	48	263	46	265	PF01095.14	Pectinesterase	93	296	298	70.72	7.00E-20	9.50E-24	1	0	CL0268
>LIPESV12_18	1	257	1	268	PF12262.3	Lipase_bact_N	1	254	268	119.16	1.80E-34	7.20E-38	1	0	
>LIPESV12-19	32	202	32	203	PF01734.17	Patatin	1	203	204	89.2	3.20E-25	4.40E-29	1	0	CL0323
>LIPESV12_20	6	219	5	232	PF01734.17	Patatin-like lipase					7.60E-22	5.10E-26	1	0	CL0323
>LIPESV12_21	27	197	27	198	PF01734.17	Patatin	1	203	204	60.78	1.60E-16	2.20E-20	1	0	CL0323
>LIPESV12_22	81	287	81	288	PF07859.8	Abhydrolase_3	1	210	211	263.24	1.40E-78	6.60E-82	1	0	CL0028
>LIPESV12_23	9	178	9	179	PF01734.17	Patatin	1	203	204	72.08	5.70E-20	3.80E-24	1	0	CL0323
>LIPESV12_24	38	257	38	258	PF12697.2	Abhydrolase_6	1	227	228	139.7	1.30E-40	9.30E-44	1	0	CL0028
>LIPESV12-25	58	131	56	132	PF03061.17	4HBT	3	78	79	38.82	7.10E-10	9.60E-14	1	0	CL0050
>LIPESV12_26	23	257	23	258	PF12697.2	Abhydrolase_6	1	227	228	148.71	2.20E-43	1.80E-46	1	0	CL0028
>C-NV12_1	51	460	51	460	PF07969.6	Amidohydro_3	1	404	404	161.59	3.20E-47	8.70E-51	1	0	CL0034
>C-NV12_2	8	282	8	283	PF04909.9	Amidohydro_2	1	271	273	102.23	3.40E-29	2.30E-33	1	0	CL0034
>C-NV12_3	241	403	229	404	PF00795.17	CN_hydrolase	12	184	186	73.61	1.20E-20	2.40E-24	1	0	
>C-NV12_4	3	175	2	175	PF00795.17	CN_hydrolase	2	186	186	88.65	2.90E-25	2.00E-29	1	0	
>C-NV12_5	14	159	6	161	PF00795.17	CN_hydrolase	12	177	186	56.52	2.10E-15	2.80E-19	1	0	

*) The conserved functional domains of each proteins were determined using Pfam (http://pfam.xfam.org/search). The best hits with highest identity and lowest Evalues were identified for each V12-enzyme via BLASTP search has been chosen for the enzyme classification. The other description for the columns please see HMMER website given in Methods.

Table S2. Bacterial biodiversity among the abhydrolase hits with lowest E-values

**Activity***	**Predicted protein**	**Phylum**	**Order**	**Genus and species**	**Evalue (BLAST-PSI)**
Lipolytic	Lipase	*Proteobacteria*	*Pseudomonales*	*Pseudomonas resinovorans*	1.00E-76
Lipolytic	esterase	*Proteobacteria*	*Alteromonadales*	*Marinobacter nanhaiticus*	4.00E-65
Lipolytic	esterase	*Proteobacteria*	Unassigned	*alpha proteobacterium* MA2	2.00E-92
Lipolytic	lipase	*Bacteroidetes*	*Cytophagales*	*Sporocytophaga myxococcoides*	5.00E-48
Lipolytic	esterase	*Verrucomicrobia*	*Verrucomicrobiales*	*Verrucomicrobium spinosum*	5.00E-22
Lipolytic	esterase	*Proteobacteria*	*Desulfarculales*	*Desulfarculus baarsii*	1.00E-52
Lipolytic	esterase	*Firmicutes*	*Thermoanaerobacterales*	*Desulfovirgula thermocuniculi*	1.00E-125
Lipolytic	esterase	*Bacteroidetes*	*Flavobacteriales*	*Flavobacterium sp.* CF136	5.00E-123
Lipolytic	Lipase	*Proteobacteria*	Unassigned	*Contendobacter odensis* Run_B_J11	7.00E-114
Lipolytic	Lipase	*Thermotogae*	*Thermotogales*	*Kosmotoga olearia*	1.00E-164
Lipolytic	esterase	*Proteobacteria*	*Rhodocyclales*	*Sulfuritalea hydrogenivorans* sk43H	1.00E-99
Lipolytic	Lipase	*Proteobacteria*	*Alteromonadales*	*Pseudoalteromonas haloplanktis*	1.00E-35
Lipolytic	esterase	*Proteobacteria*	*Rhodospirillales*	*Acidiphilium angustum*	3.00E-145
Lipolytic	Lipase	*Spirochaetes*	*Spirochaetales*	*Leptonema illini*	1.00E-99
Lipolytic	esterase	*Proteobacteria*	*Desulfarculales*	*Desulfarculus baarsii*	2.00E-81
Lipolytic	esterase	*Proteobacteria*	*Syntrophobacterales*	*Desulfomonile tiedjei*	3.00E-43
Lipolytic	CN-hydrolase	*Bacteroidetes*	*Cytophagales*	*Marivirga tractuosa*	3.00E-86
Lipolytic	CN-hydrolase	*Bacteroidetes*	*Sphingobacteriales*	*Gracilimonas tropica*	5.00E-121
Lipolytic	CN-hydrolase	*Bacteroidetes*	*Cytophagales*	*Marivirga tractuosa*	7.00E-70
Lipolytic	β-lactamase	*Proteobacteria*	*Desulfovibrionales*	*Desulfovibrio frigidus*	5.00E-150
Lipolytic	β-lactamase	*Proteobacteria*	*Alteromonadale*	*Marinobacter lipolyticus*	00.00
Lipolytic	β-lactamase	*Bacteroidetes*	Unassigned	*Prolixibacter bellariivorans*	2.00E-144
Lipolytic	β-lactamase	*Bacteroidetes*	*Flavobacteriales*	*Eudoraea adriatica*	2.00E-149
Lipolytic	β-lactamase	*Proteobacteria*	*Rhodospirillales*	*Acidiphilium angustum*	00.00
Lipolytic	β-lactamase	*Acidobacteria*	*Acidobacteriales*	*Acidobacterium capsulatum*	00.00
Dehalogenation	HAD hydrolase	*Proteobacteria*	*Alteromonadales*	*Marinobacter nanhaiticus*	1.00E-91
Dehalogenation	HAD hydrolase	*Proteobacteria*	*Desulfarculales*	*Desulfarculus baarsii*	1.00E-63
Dehalogenation	HAD hydrolase	*Acidobacteria*	*Acidobacteriales*	*Acidobacterium capsulatum*	1.00E-128
Dehalogenation	HAD hydrolase	*Acidobacteria*	*Acidobacteriales*	*Acidobacterium capsulatum*	00.00
Dehalogenation	HAD hydrolase	*Firmicutes*	*Thermoanaerobacterales*	*Thermosediminibacter oceani*	2.00E-22

*⁾ The activity was established on the basis of annotated proteins deduced from fosmid sequence data, as mentioned in the Results.

Table S3. Substrate profile and specific activity of the selected GLV12_5 purified enzyme

**GLV12_5**	Spec. act*	Opt pH	Opt T°C
α-Glucose	**0**	**n.d**	**n.d**
α-Maltose	**0**	**n.d**	**n.d**
β-Glucose	**0**	**n.d**	**n.d**
β-Cellobiose	**0**	**n.d**	**n.d**
α-Galactose	**0**	**n.d**	**n.d**
β-Galactose	**0**	**n.d**	**n.d**
α-Arabinopyranose	**2683.33±25.9**	**5.5**	**35**
β-Arabinopyranose	**0**	**n.d**	**n.d**
α-Arabinofuranose	**0**	**n.d**	**n.d**
α-Xylose	**0**	**n.d**	**n.d**
β-Xylose	**0**	**n.d**	**n.d**
β-Mannose	**0**	**n.d**	**n.d**
α-Mannose	**0**	**n.d**	**n.d**
α-Fucose	**0**	**n.d**	**n.d**
β-Lactose	**0**	**n.d**	**n.d**
α-Rhamnose	**0**	**n.d**	**n.d**

***)** The reactions were done in triplicate. Specific activity was given in µmoles *p*-NP released/min/ mg proteins. For the reactions 1 µg of purified enzyme (see Fig. S3) and 500 µg/ml (approximately 2 mM of each, see also in Methods) of respective substrates have been applied. The pH profile was ranged between 4.0 and 9.0, and the temperature profile varied between 10 to 90 °C. Only the optimal values of pH and T °C with S/D have been given in this table.

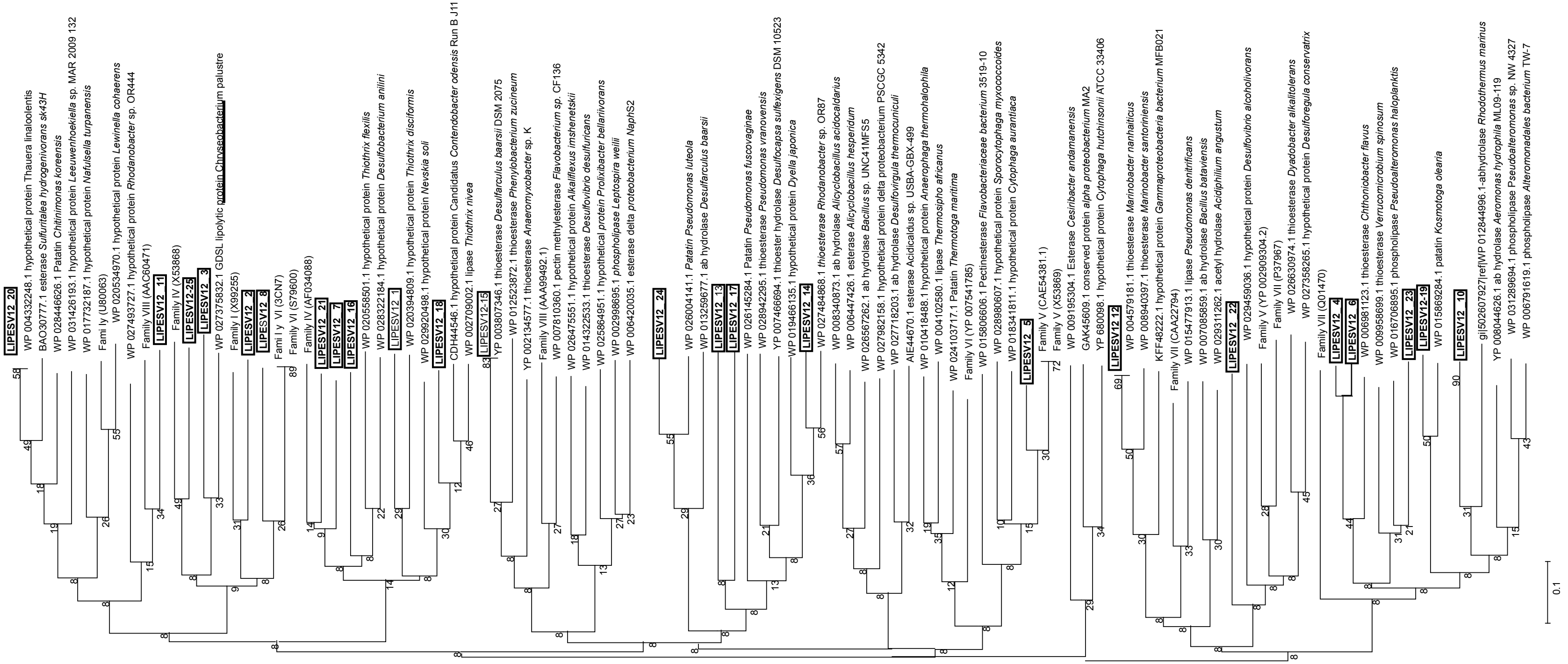

Figure S1. Neighbour-Joining trees of 24 metagenome-derived polypeptides with potential lypolytic activities (Saitou and Nei, 1987). The optimal tree with the sum of branch length = 39.55949237 is shown. The values from the bootstrap test (100 replicates) are shown at the nodes (Felselstein 1985). The evolutionary distances were computed using the JTT matrix-based method (Jones et al. 1992) and are in the units of the number of amino acid substitutions per site. All positions containing gaps and missing data were eliminated. Evolutionary analyses were conducted in MEGA6 (Tamura et al. 2013). The large phylogenetic distances between the metagenome-derived hydrolases reflect the diversity among the microbial habitats of the Vulcans' site. Among the 24 LIPESV12 proteins there are three clans of two and one clan of three polypeptides clustered very nearly one to another and to thioesterases and patatins, respectively. The LIPESV12_20 was distant to other groups and clustered with the esterase of *Sulfurtalea hydrogenivorans* sk43H.

Figure S2. Neighbor-joining phylogenetic tree of beta-lactamases related to those identified in the V12 fosmid inserts. BLAV12-enzymes clustered at separate nodes in the tree. The multiple protein alignment was conducted using the MUSCLE application (Edgar, 2004) and BioEdit software (Hall, 1999) with default settings. The phylogenetic neighbour-joining trees were constructed using MEGA v.6.06 (Tamura et al., 2013) as described in the Methods with 1000 bootstrap replicates. The scale bar reflects the number of substitutions per position. A detail description for all phylogenetic analysis is given also in the Legends to the Supplementary figures below.

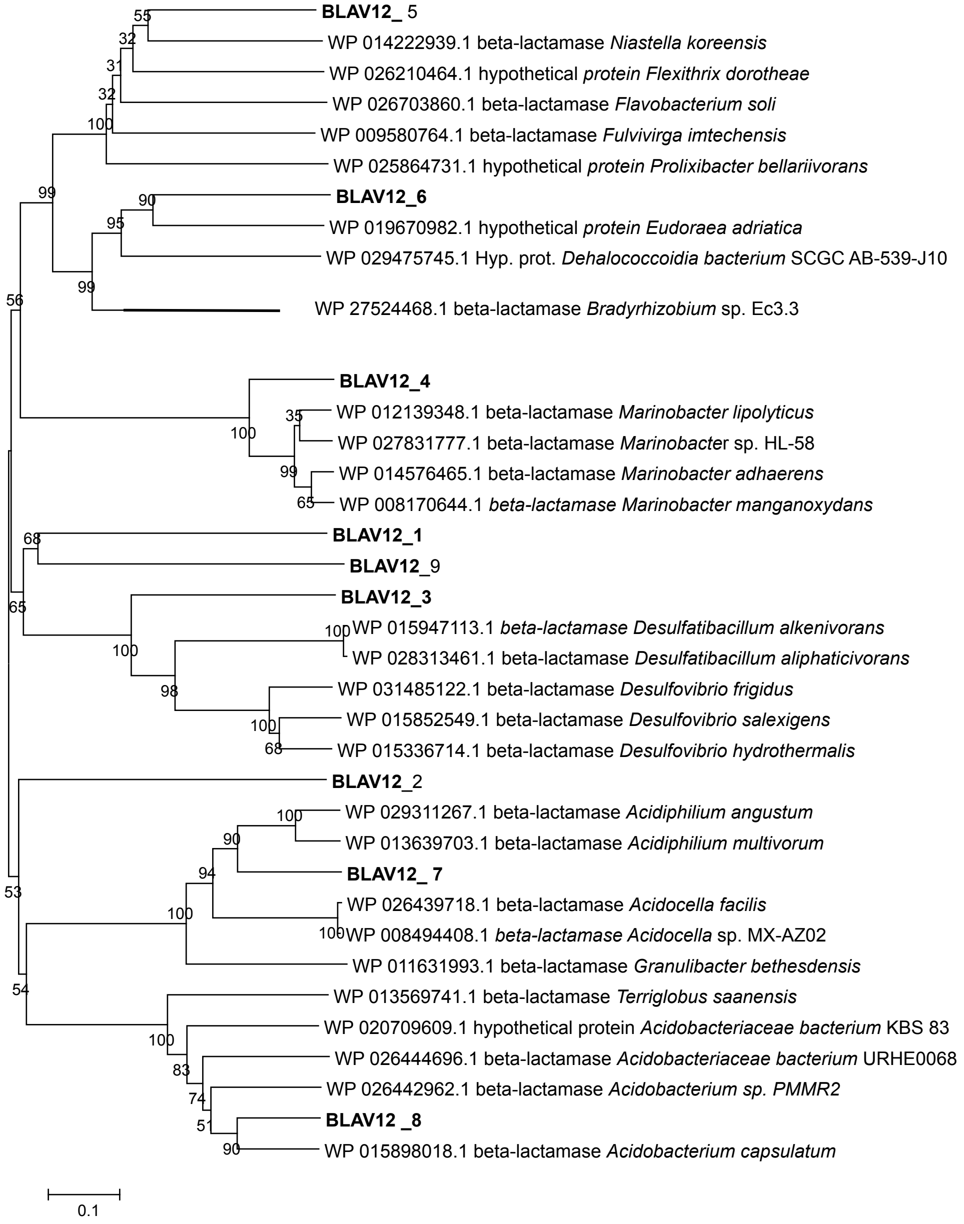

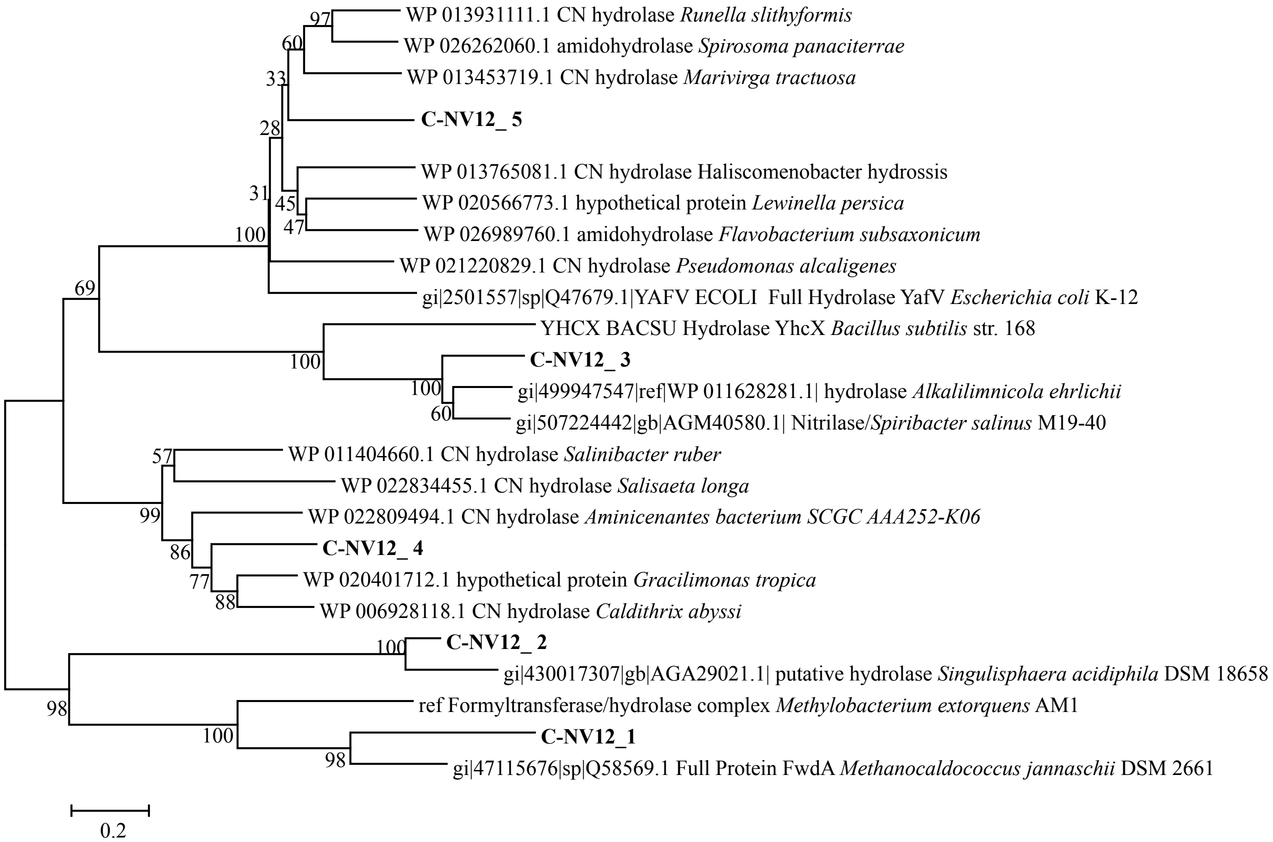

Figure S3. Positions of C-NV12-hydrolases on the neighbour-joining tree. The clustering of C-NV12_proteins with quite different groups suggests a high diversity of V12_hydrolases, and among them the C-NV12_3 was nearly clustered to the cluster of carbon-nitrogen bond hydrolase from the *Alkalilimnicola ehrlichi* and nitrilase of *Spiribacter salinus* M19-40.

Figure S4 Phylogenetic relationships between the predicted HADV12- proteins and their most related counterparts. Among the HADV12- dehalogenases the HADV12_3 and HADV12_4 showed nearest phylogenetic relationships to different trehalose phosphatases and HAD from genera of *Acidobacteriacea* and *Granullicella*, respectively. The biochemical characterization for these HADV12 enzymes are in progress.

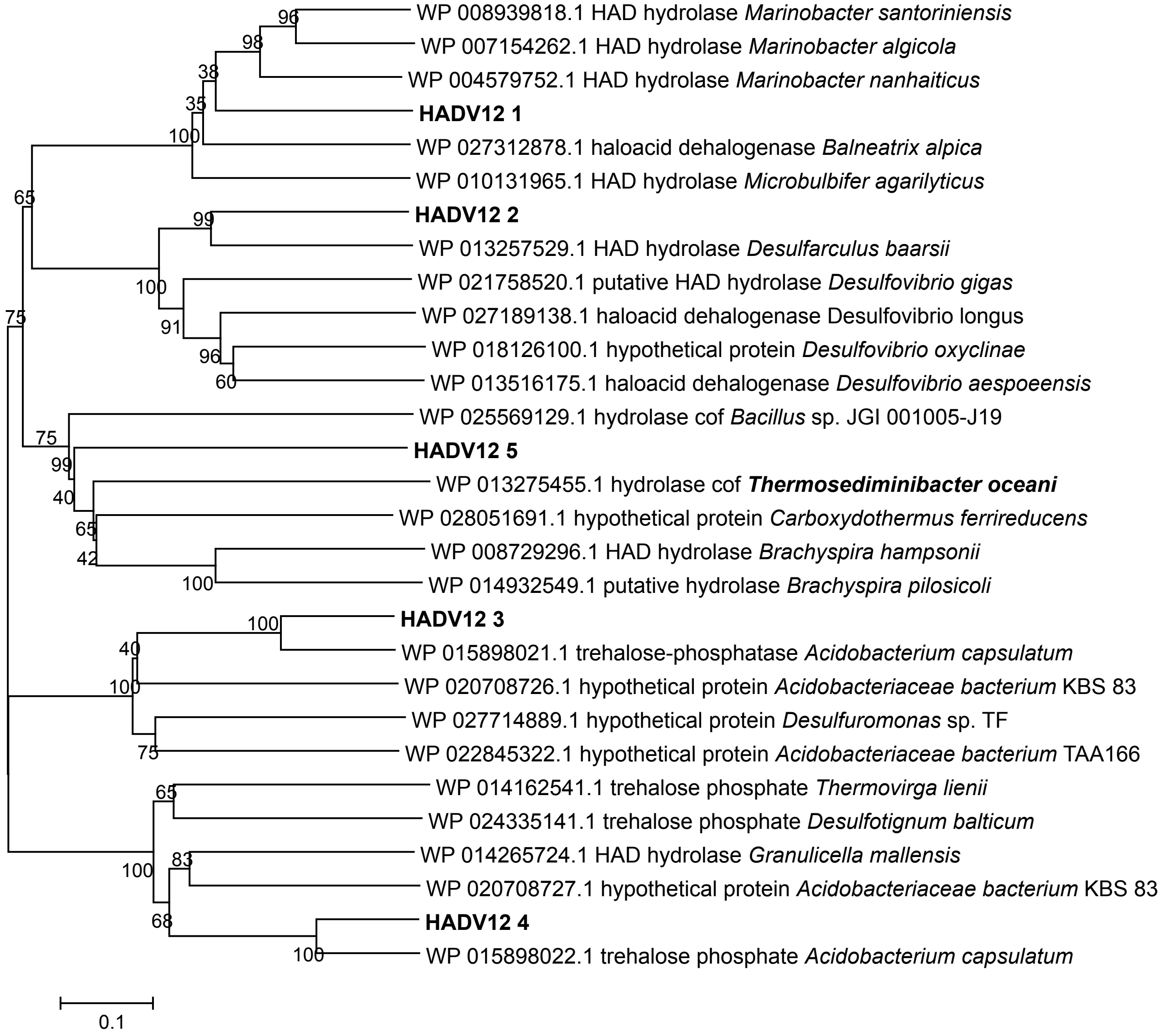

Figure S5. The phylogenetic relationships within the group of 15 predicted glycosyl hydrolases/glycosidases and their nearest homologs. Neighbour-joining tree construction procedure and bootstrap analysis was performed as indicated in the legend to the Fig. S1. Among them the GLV12_1, 2 and 16 clustered to a separate hypothetical glycoside hydrolase group, with a distinct position from other glycoside hydrolases, such as alpha-amylase from *Mahella australienensis*. Moreover, the nearest counterparts of two V12_glycoside hydrolases, where the GLV12_12 and GLV12_15 were deduced from the X-gal hits, and revealed significant homologies to beta-galactosidases of *Bacteroides xylanisolvens* (GLV12_12) and of *Catenivolum agarivorans* DS-2 (GLV12_15). The other GLV12-hydrolases have been identified among nucleotide sequences of CMC-hydrolytic hits.

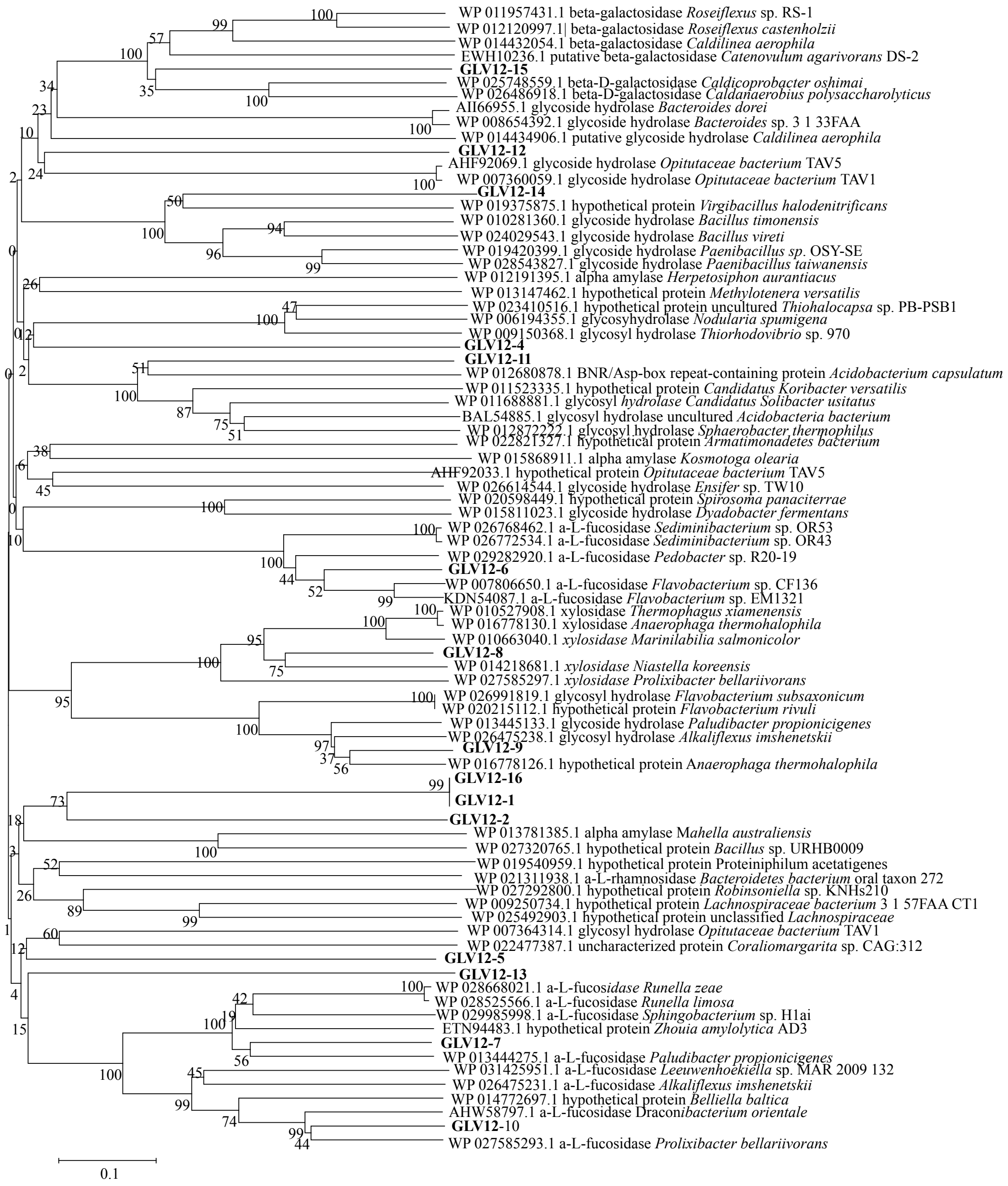

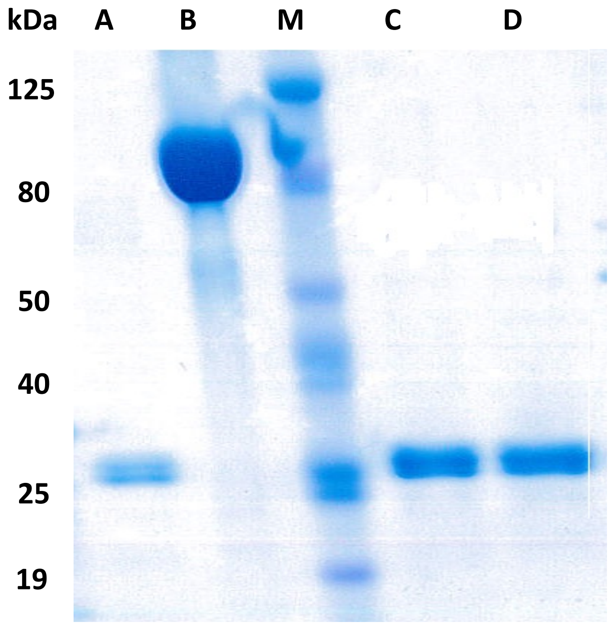

Figure S6. SDS-PAGE of the His - tagged hydrolases: LIPESV12-9 (Lane A); GLV12_5, glycosyl hydrolase(Lane B), Lane M; HyperPAGE standard (BIOLINE) with the sizes given in kDa); LIPESV12_24 (Lane C), LIPESV12_26 (Lane D), and together of are highlighted. The purified soluble his-tagged proteins revealed the expected sizes of about 30.5 kDa for LIPESV12, and 100 kDa size for GLV12_5. For the biochemical characterization only the soluble forms was applied.

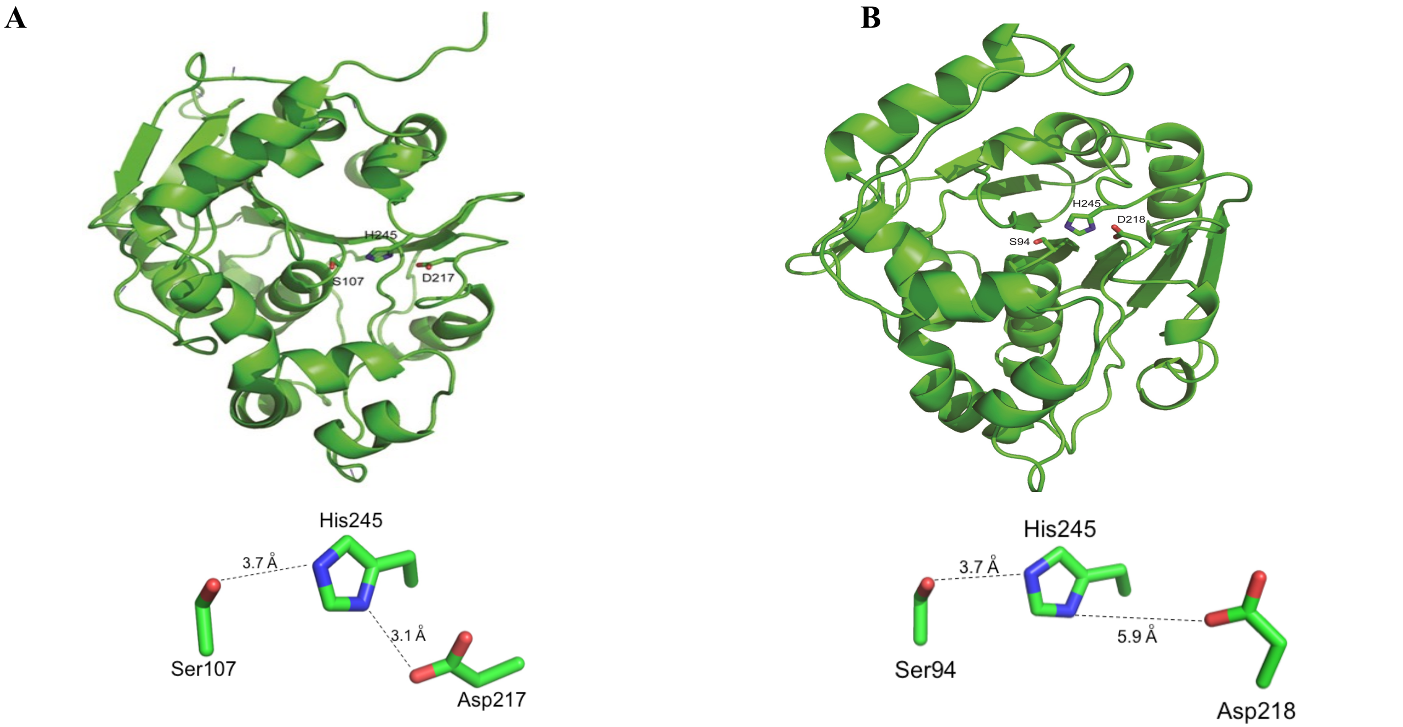

Figure S7. Predicted 3D-structures of the LIPESV12_24 **(A)** and LIPESV12_26 **(B)**. Three-dimensional models were generated using the protein fold recognition server Phyre2. Both the LIPESV12_24 and LIPESV12_26 models were produced using the structure of the murine soluble epoxide hydrolase Ephx2. (PDB code 1CR6, 16-19 % sequence identity, 100 % confidence, 95 % coverage). The Ser catalytic triads are also shown (at the bottom) with the distances between the residues. The catalytic triads are highlighted in both models. The predicted structures of the LIPESV12-proteins revealed absolute confidence (100%) and significant identity (>19 %) to their counterparts (abhydrolases) from *Chitinophaga pinensis* and *Pseudomonas aeruginosa* PAO1, which were found in the protein database PDB: C4pw0A and PDB C1cr6A/C4f0j, respectively. According to the model, all eight α-helices, as well as six ß-sheets are found in the cloned enzymes' structures. The distance between serine (S) and histidine (H) in the LIPESTV12_26 is visually longer as that of LIPESV12_24 (His/Asp distances are 3.1 Aº and 5.9 Aº, respectively). This causes, possibly, their different affinities to the long chain fatty acid esters (see the substrate affinity in the Fig. 4).
